# Lytic and genomic properties of spontaneous host-range *Kayvirus* mutants prove their suitability for upgrading phage therapeutics against staphylococci

**DOI:** 10.1038/s41598-019-41868-w

**Published:** 2019-04-02

**Authors:** Tibor Botka, Roman Pantůček, Ivana Mašlaňová, Martin Benešík, Petr Petráš, Vladislava Růžičková, Pavla Havlíčková, Marian Varga, Helena Žemličková, Ivana Koláčková, Martina Florianová, Vladislav Jakubů, Renáta Karpíšková, Jiří Doškař

**Affiliations:** 1 Department of Experimental Biology, Faculty of Science, Masaryk University, Brno, 611 37 Czech Republic; 2 National Institute of Public Health, Praha, 100 42 Czech Republic; 3 Department of Clinical Microbiology, University Hospital and Faculty of Medicine in Hradec Králové, Charles University, Hradec Králové, 500 05 Czech Republic; 4 Veterinary Research Institute, Brno, 621 00 Czech Republic

## Abstract

Lytic bacteriophages are valuable therapeutic agents against bacterial infections. There is continual effort to obtain new phages to increase the effectivity of phage preparations against emerging phage-resistant strains. Here we described the genomic diversity of spontaneous host-range mutants of kayvirus 812. Five mutant phages were isolated as rare plaques on phage-resistant *Staphylococcus aureus* strains. The host range of phage 812-derived mutants was 42% higher than the wild type, determined on a set of 186 methicillin-resistant *S. aureus* strains representing the globally circulating human and livestock-associated clones. Comparative genomics revealed that single-nucleotide polymorphisms from the parental phage 812 population were fixed in next-step mutants, mostly in genes for tail and baseplate components, and the acquired point mutations led to diverse receptor binding proteins in the phage mutants. Numerous genome changes associated with rearrangements between direct repeat motifs or intron loss were found. Alterations occurred in host-takeover and terminal genomic regions or the endolysin gene of mutants that exhibited the highest lytic activity, which implied various mechanisms of overcoming bacterial resistance. The genomic data revealed that *Kayvirus* spontaneous mutants are free from undesirable genes and their lytic properties proved their suitability for rapidly updating phage therapeutics.

## Introduction

Phage therapy is an alternative to antibiotics used against *Staphylococcus aureus* infections, especially for the treatment of chronic infections caused by multidrug-resistant strains. Virulent bacteriophages of the genus *Kayvirus* from the family *Myoviridae* are the most promising due to their high lytic activity, therefore some of them are now commercialized^1–4^. The emergence of phage-resistant bacterial strains requires innovation in phage preparations, which is usually achieved by isolating new phages from the environment^5–7^ or time-consuming procedures of phage adaptation such as phage training^8^. Upgraded preparations necessitate the safety assessments required by pharmaceutical authorities^9,10^. Phages adapted to different hosts exhibit distinct phenotypes and their specialization can be manifested as both improved infection of the host used for evolution and decreased infection of the other host types^11^. Phage and host co-evolution, driven by the resistance of bacterial hosts, results in intraspecies heterogeneity in phage genomes via emerging recombination events and single-nucleotide polymorphisms (SNPs) whose frequency and distribution vary depending on the phage and growth conditions^12,13^. The genes for host recognition, attachment, and infection are often affected by SNPs or major re-arrangements^11,14,15^. The fixation of these genome changes by natural selection enables the phages to recognize new hosts^16^ and avoid anti-phage systems^17^ such as the type II R-M system *Sau*3AI in kayviruses^18^.

Due to their polyvalence, kayviruses are valuable agents which can be used to treat infections caused by a broad range of *S. aureus* and even non-*S. aureus* staphylococcal species^1,3,7^. Bacteriophage K is the best studied member of the genus *Kayvirus*^18,19^. The unit genomes of kayviruses are about 140 kb in size with more than 200 genes, as was reviewed by Łobocka *et al*.^20^. Early expressed host-takeover genes are located in identical left and right long terminal repeats (L-LTR and R-LTR, respectively), in which R-LTR forms genomic redundancy^21–23^. The majority of *Kayvirus* genomes contain group I introns, often encoding a homing endonuclease, which are located in the genes for DNA metabolism, morphogenesis and lysis^24–26^.

In our previous studies, we isolated almost twenty mutants of kayvirus 812, which, when used in cocktails, extended the amount of susceptible *S. aureus* strains of different origin^1,27,28^. Phage 812 was later studied at the proteomic and structural level^29,30^, however detailed genomic characterization has not been performed yet. Here, spontaneous mutants of phage 812 exhibiting a broad host range on recently circulating methicillin-resistant *Staphylococcus aureus* (MRSA) strains were isolated. With respect to interest in using the mutants in phage therapy, we focused on deep sequence analysis of their genomes, characterization of genomic variations, and exclusion of potential risks for phage therapy as required by pharmaceuticals authorities^31^.

## Results

### The host range of phage 812 and its mutants

**Figure 1:**
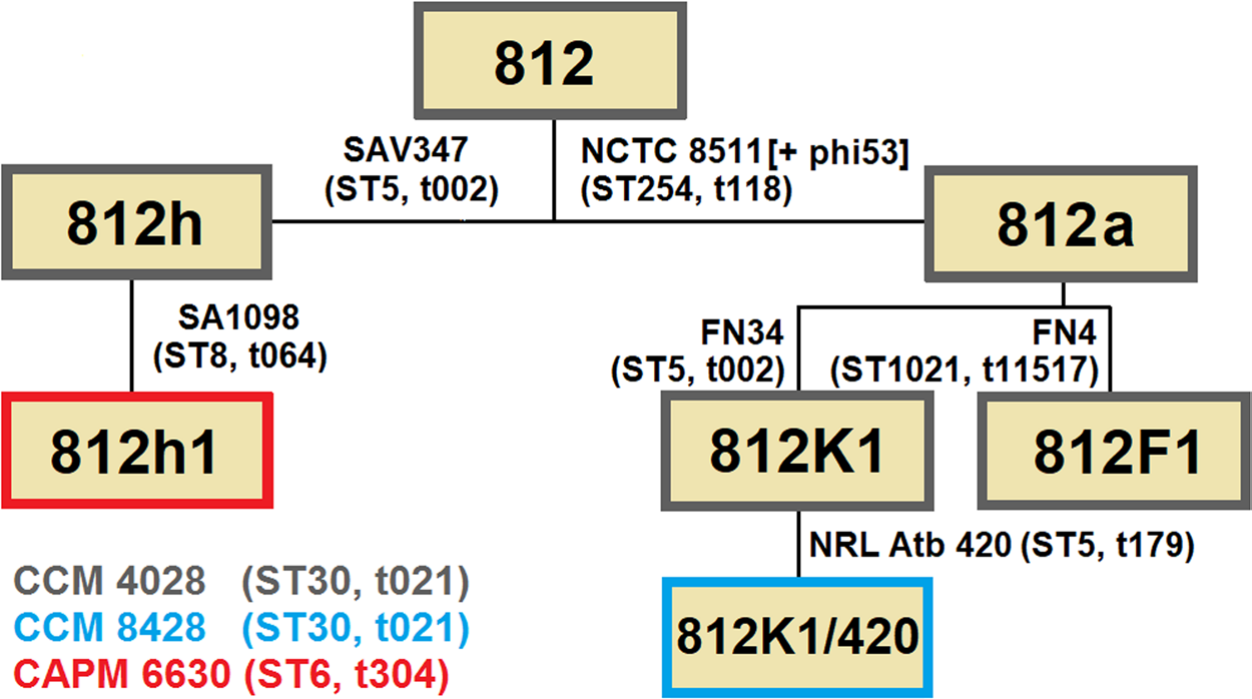
Diagram showing the isolation procedure of spontaneous host-range mutants of phage 812 represented by boxes. All phage mutants were isolated from a single plaque on selection *S. aureus* strains shown next to the corresponding mutants. Phage mutants were then propagated on *S. aureus* strains that are colour coded according to the phage mutant. The sequence type (ST) and the staphylococcal protein A (*spa*)-type of each strain are given in parentheses.

Mutants of phage 812 exhibiting a broader host range were isolated as rare plaques (with frequencies 10^−6^–10^−9^) on various *S. aureus* strains resistant to the parental phage (Fig. 1). The mutant phage 812a was a starting phage for several subsequent multi-step mutants isolated on different strains (Fig. 1). As the phage 812a-derived mutants did not exhibit a satisfactory lytic effect on livestock-associated MRSA (LA-MRSA) strains (Supplementary Table S1), a new lineage of mutants represented by phage 812h1 was selected from wild-type phage 812 on phage-resistant LA-MRSA strains (Fig. 1).

**Figure 2:**
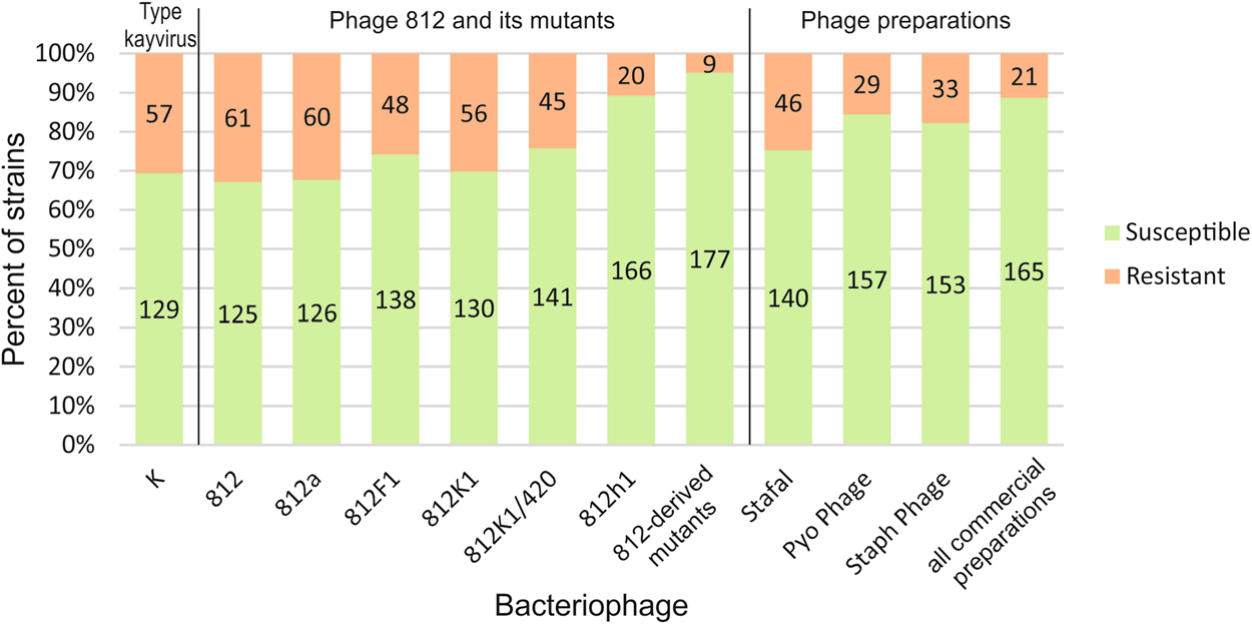
Lytic activity of phages K, 812 and its mutants, and kayviruses from commercial preparations Stafal (Bohemia Pharmaceuticals, Czech Republic), PyoPhage (Eliava Biopreparations, Georgia) and StaphPhage (Microgen, Russia) determined on a set of 186 methicillin-resistant *S. aureus* strains. The number of strains susceptible or resistant to a given phage is shown in each column. The susceptibility patterns of strains are listed in Supplementary Table S1. Susceptibility of strains to at least one of the 812-derived mutants and at least one of the commercial preparations is also shown.

To evaluate the efficiency of the phage mutants, their lytic ability was tested on a set of well-characterized MRSA isolates (n = 186) and compared with type phage K and kayviruses isolated from commercial therapeutic preparations Stafal (Bohemia Pharmaceuticals, Czech Republic), PyoPhage (Eliava Biopreparations, Georgia) and StaphPhage (Microgen, Russia). The tested *S. aureus* strains belonged to 18 different sequence types (ST) and 45 staphylococcal protein A gene (*spa*)-types chosen to represent the major global MRSA clones (Supplementary Table S1). The susceptibility to phages varied among the strains of the same ST and even of the same *spa*-type. Of the tested strains, 59.1% (n = 110) were susceptible to all the tested bacteriophages. In contrast, 4.8% (n = 9) of isolates, mostly of ST45, were completely resistant to all the tested phages (Fig. 2, Supplementary Table S1). Overall, the number of tested strains lysed by wild-type phage 812 or at least one of its mutants (n = 177) was higher than phages from commercial preparations Stafal (Bohemia Pharmaceuticals, Czech Republic), PyoPhage (Eliava Biopreparations, Georgia), and StaphPhage (Microgen, Russia) (n = 165) (Supplementary Table S1). The mutant 812h1 exhibited the broadest host range, lysing almost 90% (n = 166) of the strains in total (Fig. 2). Nevertheless, 20 strains remained resistant to this mutant, but 11 of them were susceptible to some other phage 812-derived mutant, mostly to 812K1/420 (Supplementary Table S1).

### Genomic analysis of phage 812 and its mutants

**Figure 3:**
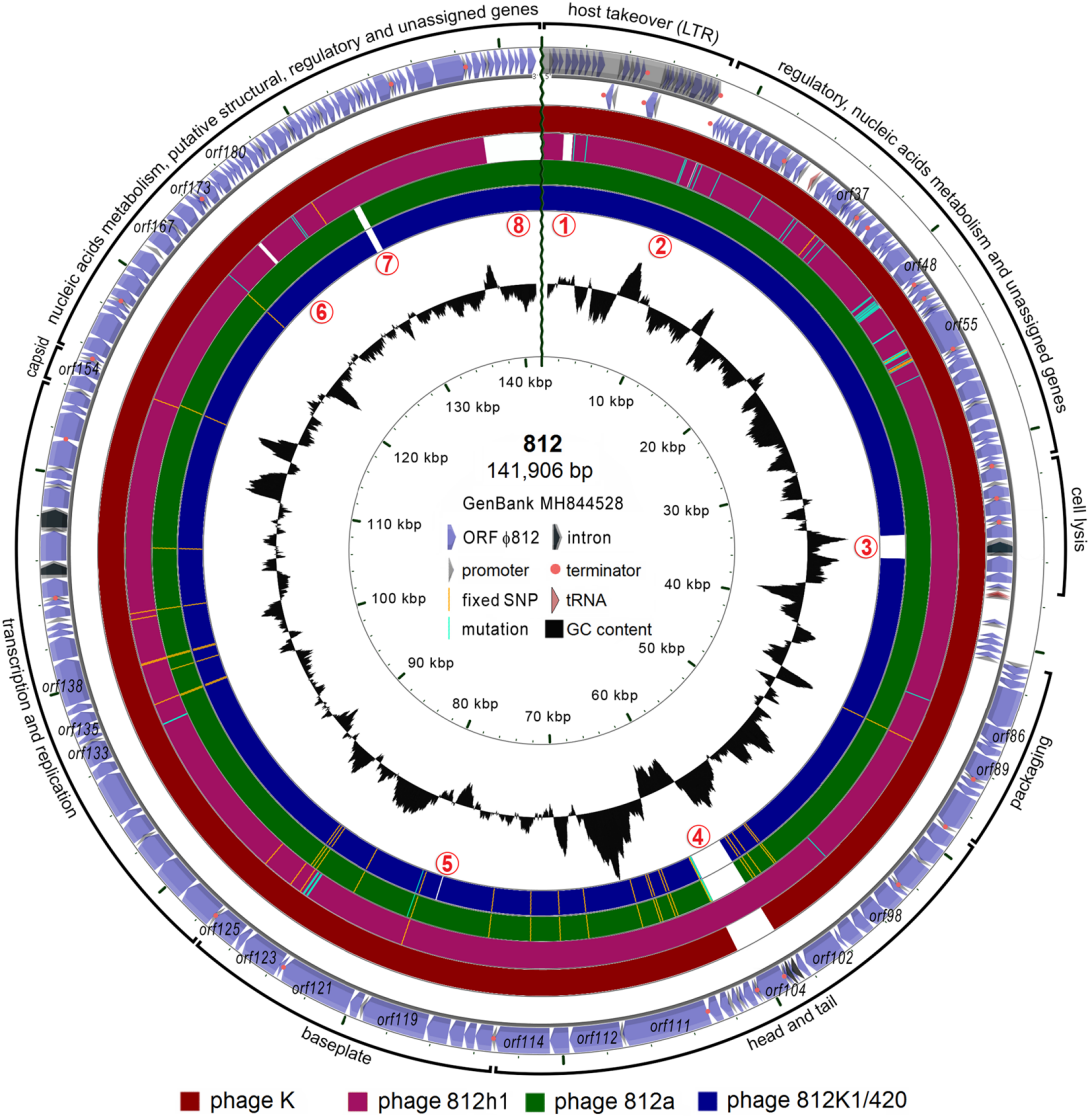
Phage 812 unit genome (GenBank accession number MH844528) and its comparison with phage K (NC_005880), phage mutant 812h1 (MH844529), phage mutant 812a (KJ206560), and 812a-derived mutant 812K1/420 (KJ206563). The major differences are labeled by red numbers: (1) host-takeover region showing low similarity between 812 and 812h1; (2) *orf23* and *orf24* of 812h1 that were not found in phage 812; (3) *orf73* encoding endolysin (LysK) that contains a partial deletion in 812a-derived mutant 812K1/420; (4) *orf103* for a tail tube protein that does not contain non-coding introns in 812a-derived mutants, and *orf104* for a putative intron-encoded nuclease which is missing in phage K and 812a-derived mutants; (5) *orf119* for tail fibre complex that contains a partial deletion in 812K1/420; (6) *orf173* for putative membrane-associated protein which is split into two open reading frames in 812h1 due to a partial deletion; (7) *orf190* and *orf191* deleted in all 812a-derived mutants, which led to fusion of *orf189* for regulatory protein and *orf192* for putative membrane protein; and (8) terminal part of the genome which is highly different from 812h1. Mutations and fixed polymorphisms with impact on protein sequences in compared phages are shown. The genome comparison is based on tblastx with an identity cut-off of 95%.

**Figure 4:**
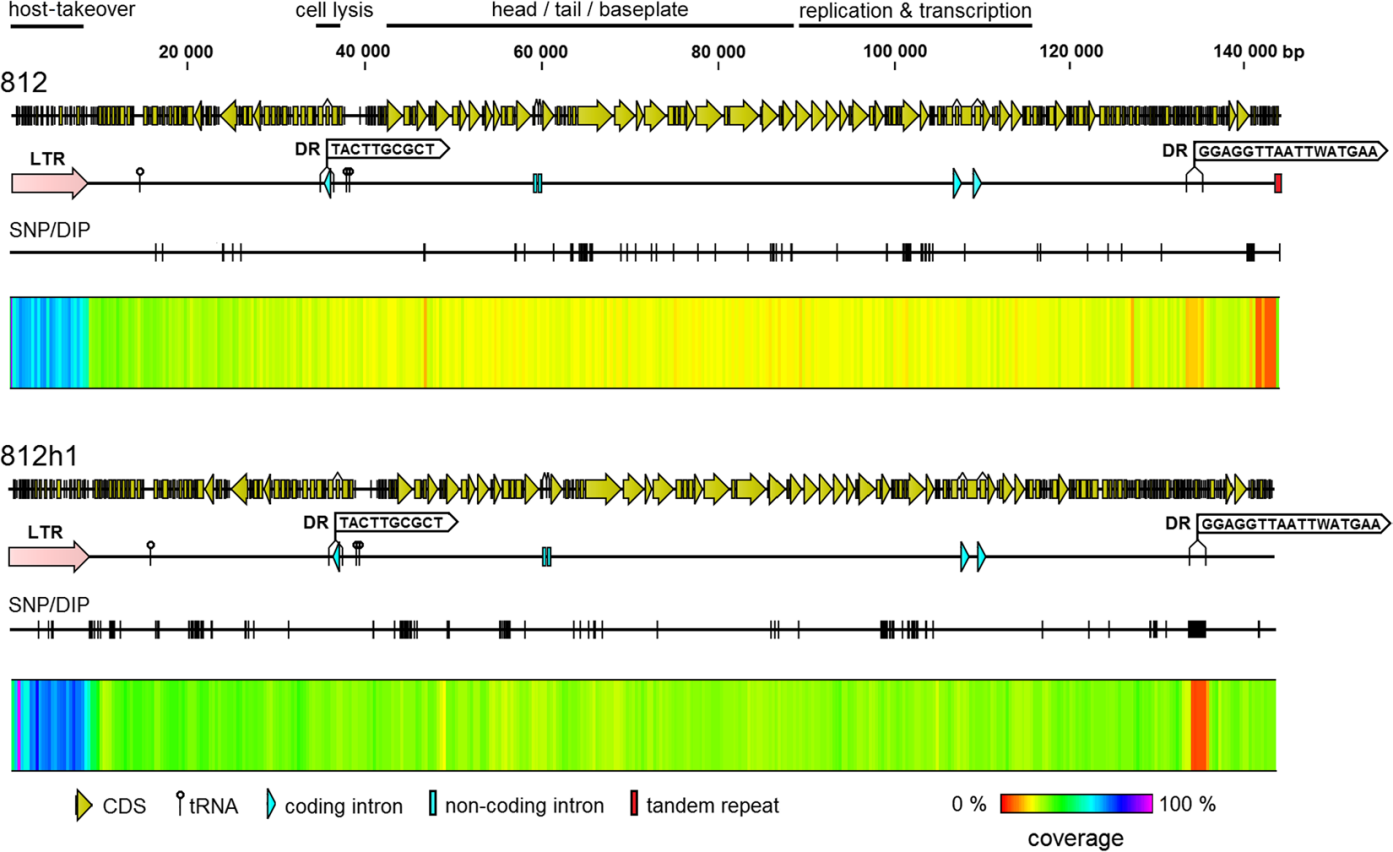
Coverage differences of genomic regions achieved by mapping of sequencing reads to unit genomes of phages 812 and 812h1. Long terminal repeats (LTRs) were delimited according to excessively increased coverage at the left end of the genomes due to the alignment of reads of both the LTRs. The increased coverage at the right end of the phage 812 genome is caused by a variable number of 35-bp tandem repeats. The genome annotations are illustrated on three feature lines: the top line shows coding sequences; the middle line indicates the location of LTR, tRNA genes, introns, and the direct repeats (DR) serving as recombination sites in the endolysin gene of phages 812F1 and 812K1/420, and 2-kb deletion/insertion polymorphism in phage 812h1; and the bottom lines show the positions of all polymorphisms.

**Figure 5:**
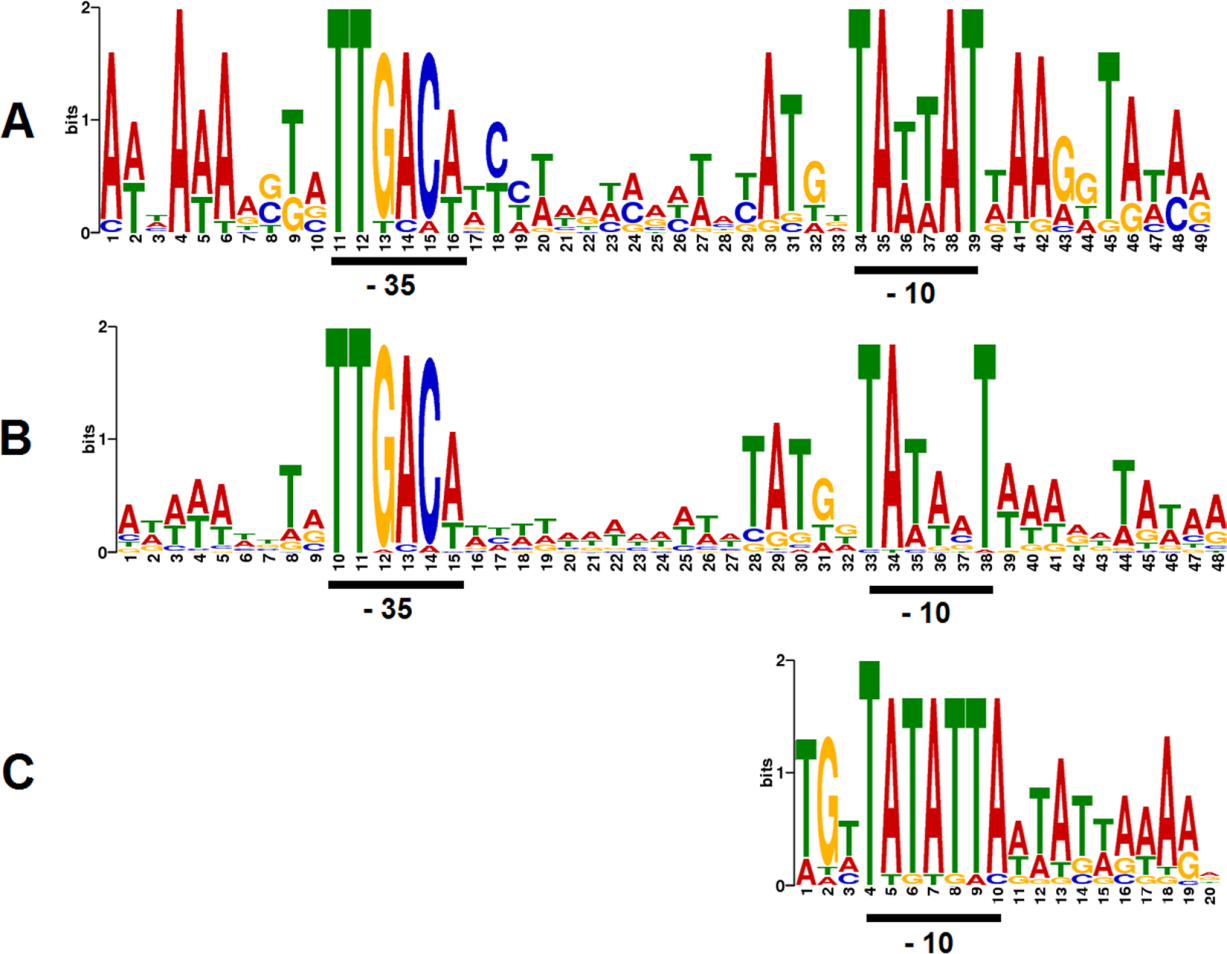
Sequence logos of phage 812 promoters. (**A**) Bacterial promoters in long terminal repeats contain typical −35 and −10 sequences. (**B**) Putative early promoters recognised by host sigma factor. (**C**) Putative late promoters recognised by phage sigma factor. The late promoters drive the transcription of genes for homing endonuclease in long terminal repeat and in the polymerase gene intron, and genes for packaging, the structure of the head, tail and baseplate, and lysis. No conserved −35 sequence was found in phage late promoters.

The phage 812 unit genome, 141,906 bp in size with a GC content of 30.4%, starts with a 8,484-bp left long terminal repeat (L-LTR) and ends with a tandem repeat with a 35–bp-long repeat unit sequence TATTAYTACTACTAAGTACCTTTGTTATGTACTAC (Figs 3 and 4). The copy number of the repeat unit is variable, ranging from four to more than twenty complete copies in the phage 812 population and down to two copies in the mutant 812K1/420 (Supplementary Fig. S1). The right LTR (R-LTR), identical to L-LTR, comes after the unit genome and forms the terminal redundancy (Fig. 4). In total, 218 coding sequences (CDS), 63 putative promoters, and 30 putative intrinsic terminators were predicted in the unit genome (Figs 3 and 5). Three genes for tRNA (tRNA-Met-CAT, tRNA-Phe-GAA, and tRNA-Asp-GTC) and one gene for pseudo tRNA with an unknown function were predicted in the phage 812 genome. At least five introns were detected, of which three encode putative endonucleases, as in phage K^23,32^. The genomes of phage 812 and phage K exhibit 98.5% identity; although phage 812 contains two additional non-coding introns in the tail tube protein gene and the adjacent gene for putative endonuclease, which were also localized in phage 812h1 (Fig. 3) and in four previously described kayviruses, i.e. A3R, 676Z, Fi200W, and P4W^20^.

In total, 176 variations were identified in the sequencing data of the wild-type phage 812 genome (Supplementary Table S2): 171 single-nucleotide polymorphisms (SNPs) and 5 deletion/insertion polymorphisms (DIPs), often localized in host range-associated genes (Fig. 4, Supplementary Table S2). A lot of minor SNPs and DIPs with a frequency in the genome < 50% of wild-type phage 812 were found to predominate in the mutant 812a and all its derivatives 812F1, 812K1, and 812K1/420 (Table 1). Such polymorphisms are called “fixed” in the text below and some of them affected encoded proteins (Fig. 3).

**Table 1:** Single-nucleotide polymorphisms (SNP) and deletion/insertion polymorphisms (DIP) in phage 812 genome fixed in 812a and its derived mutants 812F1, 812K1, and 812K1/420 and new mutations occurred in 812a. Only genome changes with impact on protein sequences are listed.

ORF of phage 812	Size (nt)	Gene product	Proposed function	Number of fixed SNP or DIP	Mutations in 812a	Number of amino-acid substitutions (sub) and insertions (ins)
*orf89*	1188	membrane-associated protein	packaging	1	—	2 ins
*orf102*	1764	tail sheath protein	genome ejection	5	—	5 sub
*orf103*	426	tail tube protein	genome ejection	0	intron loss	1 sub, 1 ins
*orf104*	1404	putative intron nuclease	intron homing	—	gene deletion	
*orf105*	141	hypothetical protein	tail assembly	4	2 nt sub	3 sub
*orf110*	537	tail morphogenetic protein	tail assembly	6	—	2 sub
*orf111*	4059	tape measure protein	tail assembly	20	—	6 sub
*orf112*	2427	tail murein hydrolase	penetration	1	—	1 sub
*orf113*	888	cysteine protease	penetration	1	—	1 sub
*orf114*	2547	tail central spike	penetration	1	—	1 sub
*orf116*	525	baseplate component	adsorption	1	—	1 sub
*orf119*	3060	tail fiber complex	adsorption	1	1 nt sub	2 sub^1^
*orf121*	3459	adsorption-associated protein	adsorption	1	—	1 sub
*orf123*	1923	receptor-binding protein	adsorption	3	—	3 sub
*orf135*	453	hypothetical protein	DNA metabolism	2	—	2 sub
*orf138*	2115	ribonucleotide reductase	DNA metabolism	6	—	3 sub
*orf141*	321	oxidoreductase	DNA metabolism	2	—	1 sub
*orf144*	3219	DNA polymerase	DNA metabolism	1	—	1 sub
*orf154*	522	putative capsid component	head assembly	1	—	1 sub
*orf167*	459	putative structural protein	virion assembly	1	—	1 sub
*orf189*	294	putative regulatory protein	regulation	0	456-nt deletion	fusion of *orf189* and *orf192*
*orf190*	288	membrane-associated protein	regulation	0		
*orf191*	117	putative transcription factor	regulation	0		
*orf192*	264	membrane-associated protein	regulation	0		

^1^In phage 812K1/420 a premature stop-codon, due to a 77-nt deletion and 1-nt substitution, split the gene.

In addition, a few gene product alterations specific for each of the multi-step mutants were found. Phage 812K1 differs from the others by an 11-aa intrinsic deletion in an encoded ligase and 78-aa deletion along with the substitution of 4 aa in an encoded primase. Both the phages 812F1 and 812K1/420 encode truncated endolysin (a 211-aa deletion) due to a 1509-bp deletion in the endolysin gene as reported previously^26^. The gene for the tail fibre protein complex (*orf119*) of the phage 812K1/420 contains a premature stop codon due to a 77-bp deletion that divides this gene into 2 ORFs. Another premature stop codon caused by a nonsense mutation divides the gene for ribonuclease H of phage 812K1/420 into two ORFs without changing the reading frame.

### Genomic analysis of phage 812h1

The phage 812h1 was selected in two steps using two distinct strains (Fig. 1). The unit genome of phage 812h1 is 142,150 bp long with an 8,432-bp-long L-LTR. The unit genome completely lacks the tandem repeat locus typical for phage 812, 812a and its subsequent mutants, as well as for phage K (Fig. 4 and Supplementary Fig. S1). In total, 219 CDS, 62 putative promoters, 31 putative intrinsic terminators, 3 tRNA genes and 1 pseudo tRNA gene were predicted in the unit genome. 255 SNPs and 5 DIPs were identified mostly in the genes for structural proteins and replication, as in phage 812 (Supplementary Tables S2 and S3). On the other hand, many SNPs of phage 812h1 are located in the genes for terminal repeat-encoded proteins (Fig. 4, Supplementary Table S3). One of the most apparent variabilities is the duplication of the gene for terminal repeat-encoded protein (*orf7*) located in the host-takeover region. The *orf7* duplication is bordered by the nucleotide motif TAGGAT, which is present in more than 90 copies in all *Kayvirus* genomes. The next apparent DIP is for a 2-kb-long sequence including *orf195* – *orf200* of unknown function, bordered by a unique direct repeat GGAGGTTAATTWATGAA (Fig. 4). The phage 812h1 genome differed from wild-type phage 812 and all mutants derived from phage 812a in multiple loci (Fig. 3, Supplementary Table S4).

**Figure 6:**
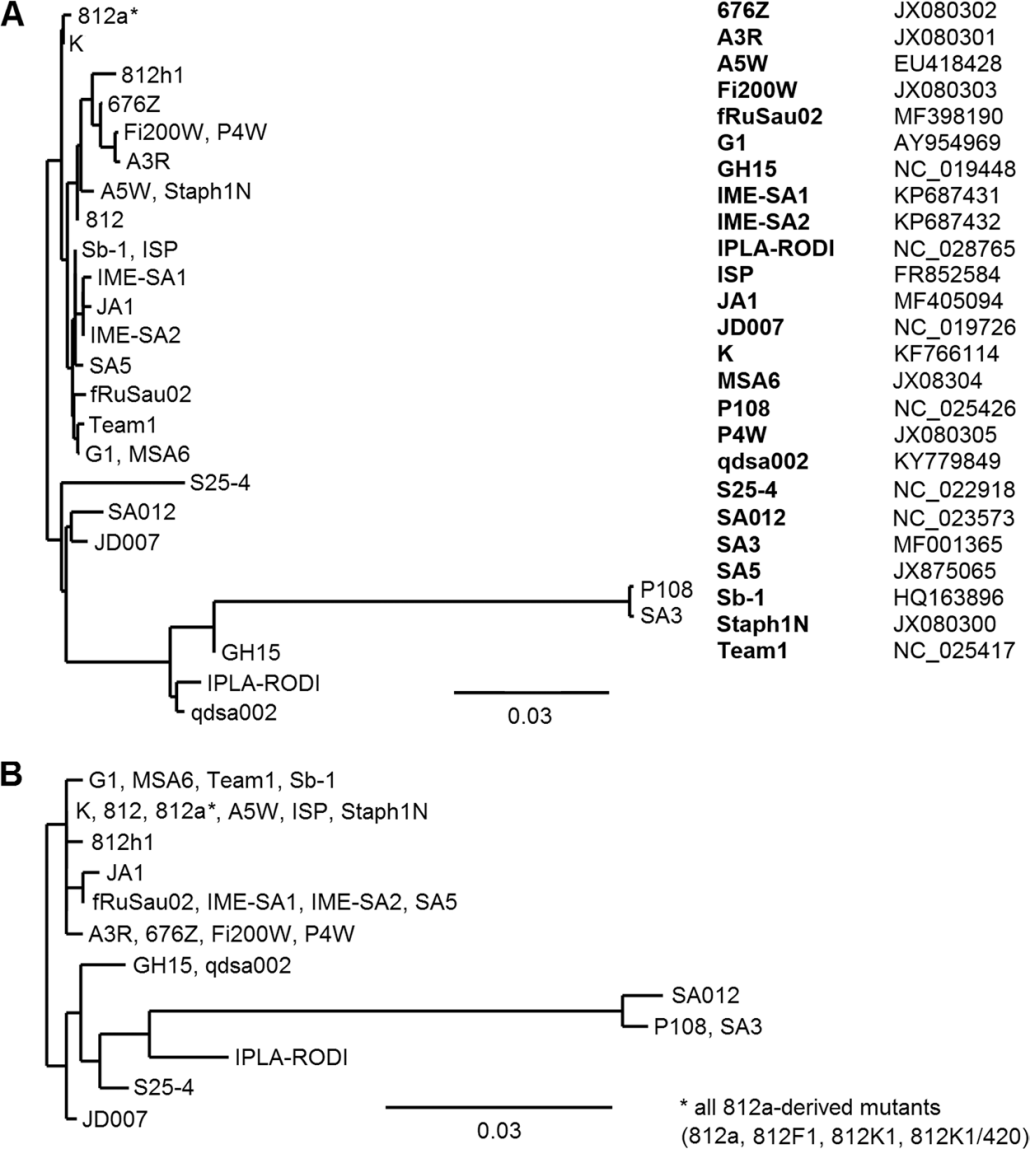
Unrooted phylogenetic trees showing similarity of two *Kayvirus* receptor binding proteins homologous to (**A**) protein encoded by phage 812 *orf123* and (**B**) protein encoded by phage 812 *orf125*. The phylogenetic trees were reconstructed using the maximum likelihood method (aLRT test) performed on the Phylogeny.fr platform. GenBank accession numbers of the compared phage genomes are listed next to the trees. The trees are drawn to scale, with branch lengths measured in the number of substitutions per site.

Two different types of genes encoding receptor binding proteins (RBPs) corresponding to phage 812 *orf123* and *orf125* determine the host range of kayviruses through sequence variability and by the adsorption mechanism that may vary even in closely related phages^29,33,34^. These genes exhibit a high variability among *Kayvirus* genomes (Fig. 6), which could help the phages to adapt to a new host strain. The phages 812 and 812h1 differ in both the RBPs (Supplementary Table S4) and by SNPs content in both RBP-encoding genes (Supplementary Tables S2 and S3). The 812a-derived mutants only fixed SNPs of the parental phage 812 in the *orf123* homologue (Table 1).

### Safety assessment

Because of the extensive presence of variable genetic elements and virulence or resistance genes in the genome of *S. aureus* strains used for the selection and propagation of the phage 812-derived mutants, it was necessary to check the phage genomes for the possible acquisition of genes with potential safety concerns. None of the isolated phage mutants carried genes for antibiotic resistance, bacterial virulence factors, or mobile elements-derived sequences. Similarly, no acquisition of the genes for lysogeny was found in the analysed phage genomes.

To exclude the possibility of generalized transduction, the frequency of packaging of the genes *mecA*, *tetK* and *blaZ* (encoding resistance to methicillin, tetracycline, and penicillin, respectively) into phage particles was analysed by relative quantification (Supplementary Table S5). Kayviruses K, and 812K1/420 as a representative of 812-derived phages, and PyoPhage was propagated on well-characterized MRSA strains: the monolysogenic strain COL with plasmid pT181 (*tetK*) and double lysogenic strain 08/019 of a USA300 clone with penicillinase plasmid pUSA-Houmr-like (*blaZ*). The restriction-deficient prophage-less methicillin-susceptible *S. aureus* (MSSA) strain RN4220 (pT181 and pUSA-Houmr-like) was used to prove the absence of spontaneously induced prophages from the COL and USA300 strains. Compared to simultaneously tested transducing siphovirus 80α, the resistance gene packaging by the studied kayviruses was 1000-fold lower (Supplementary Table S5).

## Discussion

As shown in this study, the isolation of host-range spontaneous mutants is another and effective way to obtain improved therapeutic phages against almost every *S. aureus* strain. The strains exhibited distinctive phage susceptibility patterns (Supplementary Table S1), which indicates the action of several anti-phage defence mechanisms. This indicates that some of the mechanisms are shared among the strains of different genotypes, as was demonstrated e.g. for strains susceptible only to phage mutants with a truncated endolysin gene^26^. Since there is no apparent association between the strain genotype and the phage susceptibility, it can be assumed that anti-phage defence mechanisms are mainly encoded by the accessory genome. To overcome various defence mechanisms, it seems advantageous to use phage cocktails or apply the phages sequentially during the phage therapy.

Compared to the parental phage, all the isolated mutants exhibited point mutations and/or larger genomic rearrangements, some of which were reported before^27^. Deep sequencing analysis of wild-type phage 812 and the most effective mutant 812h1 showed that numerous genomic polymorphisms emerged during the phage propagation. In agreement with the recent evolutionary study^13^, most of the SNPs accumulated in genes with an assumed effect on phage fitness.

Because of the different history of isolation and various genotypes of selection and propagation strains, the conditions for SNP accumulation in the analysed phages were different. The number of SNPs in both the wild-type phage 812 and recently isolated phage mutant 812h1 was higher than reported in *Pseudomonas* phages^13^. The high prevalence of polymorphisms indicates a high mutation rate that enables the diversification of the phage populations. In the genomes of 812a-derived phages obtained by 454-sequencing technology, a deep sequence analysis was not possible, so only the SNP/DIP fixation was evaluated. The procedure for isolating a phage mutant effective against to-date resistant strains were based on natural selection on the phage-resistant strain and the random picking up of a rare single plaque which may lead to genetic drift. Therefore, the understanding of evolutionary processes in natural *Kayvirus* populations requires further study, including the deep sequence analysis of isolates with a unified history.

Sequences of 812h1 distinct from the wild-type phage 812 were found in the genomes of related phages ISP, vB_SauM-fRuSau02, Fi200W, MSA6 or G1^2,3,20,32^. Some of the sequences were also found in wild-type phage 812 sequencing data, but with very low coverage. This suggests that a large pool of phage sequences is maintained in minor genome variants persisting in *Kayvirus* populations.

Numerous direct repeat motifs found across the genomes of kayviruses enable genomic rearrangements, therefore in the phage progeny, the polymorphic loci can be detected in different combinations, which plays an important role in phage diversification. Propagation on strains with different genotypes can cause fixation of the particular polymorphisms beneficial for phage fitness, as was reported in streptococcal phages able to avoid host defence mechanisms^17^. Another repeat-associated change in phage 812-derived mutants is of the copy number of tandem repeats in the variable right terminus as a characteristic feature of *Kayvirus* genomes, which probably plays a significant role in replication and packaging^35^.

Compared to phage 812, host-range mutant 812h1 exhibited differences in both the genes for RBPs, while in the 812a-derived mutants they were only in one of them (*orf123*). As recently described for bacteriophage SA012, RBP encoded by a gene homologous to *orf125* of phage 812 seems to play an essential role in binding onto the backbone of wall teichoic acids (WTA); the additional binding of RBP encoded by a gene homologous to *orf123* of phage 812 to N-acetyl-α-D-glucosamine (α-GlcNAc) of WTA is required for the effective infection of some phages^34^. In kayviruses, the homologs of the α-GlcNAc-binding RBPs exhibit higher variability than WTA-backbone-binding RBPs, which indicates that they determine the host specificity.

Phages suitable for therapy should not contain genes for the lysogenic life cycle, such as genes for integrases and those for virulence or drug resistance factors, which could be horizontally transferred into the genome of the bacterial host^8,9,31^. A recent comparison of the complete genomes of myoviruses, including 14 kayviruses, proved that they are all strictly lytic and do not carry antibiotic resistance or bacterial virulence genes^36^. Except for the non-spontaneous T4 mutants^37,38^, the potential of bacterial gene transduction has not been reported for the strictly virulent phages, some of which even break down the bacterial DNA to generate the building blocks required for the synthesis of progeny DNA^39^. The genomes of these phages feature specific gene products involved in nucleotide metabolism, such as ribonucleotide reductase subunits. It has been shown that the genes for Nrd are host-like auxiliary metabolic genes promoting phage replication^40^. Importantly, the above safety requirements hold for phage 812 and all its host-range mutants described in this paper. Therefore, a shorter assessment time frame, similar to that of the influenza vaccine, should be considered with regard to the data which indicate that bacteriophage strain replacement or addition has no impact on the safety of the preparation^10^.

The results of our study help to clarify the molecular basis of differences in phage lytic properties. These findings also contribute to a better understanding of *Kayvirus* genomics and to establishing the rules for updating phage preparations. During commercial phage production, confirmation of the phage identity including its genome consistency is required from the master seed lots to the final products^9^. Since the genomic sequences of host-range phage mutants are almost identical, sequencing PCR amplicons of the most variable regions could distinguish the phages in commercial products from different companies. Alternatively, phage mutants can be distinguished using a multiplex PCR assay designed to target sequences specific for individual phage mutants (Supplementary Fig. S2). Nevertheless, it is necessary to determine the level of acceptable genome variability between the phage master seed lots and the final products. Our results show that the emergence of genomic alterations in therapeutic phages is a naturally occurring process beneficial for rapidly gaining new host-range mutants. Importantly, we showed that the phage mutants have no undesirable gene acquisitions with impact on the safety of phage therapeutics.

## Materials and Methods

### Bacterial strains

186 MRSA strains of various origin (Supplementary Table 1) were obtained from the National Reference Laboratory for Antibiotics and National Reference Laboratory for Staphylococci (National Institute of Public Health, Prague, Czech Republic), University Hospital Brno (Czech Republic), and farm livestock and the environment from different locations in the Czech Republic, deposited in the collection of the Bacteriology Department (Veterinary Research Institute, Brno, Czech Republic). Propagating *S. aureus* strains CCM 4028 and CCM 8428 were provided by the Czech Collection of Microorganisms (Masaryk University, Brno, Czech Republic). Strain RN4220 was obtained from Prof. F. Götz (University of Tübingen, Germany). Strain COL was kindly provided by Prof. Hermínia de Lencastre (Universidade Nova de Lisboa, Portugal and The Rockefeller University, New York, NY). Other propagating and selection strains were isolated in this or earlier studies^1^ and are genotypically characterized below. Propagation strain CAPM 6630 for phage 812h1 was deposited in the Collection of Animal Pathogenic Microorganisms (Veterinary Research Institute, Brno, Czech Republic).

### Genotyping of *S. aureus* strains

All strains were genotypically characterized using multi-locus sequence typing (MLST) according to Enright *et al*.^41^ and *spa*-typing according to the Ridom StaphType standard protocol (www.ridom.org). The software package Ridom StaphType v2.2.1 (Ridom, Germany) was used for *spa*-type assignment. Clonal complex analysis was performed with eBURST v3^42^. All strains were tested for the *mecA* gene as described previously^43^. The prophage content in propagating and selection strains was investigated by multiplex PCR assay^44^.

### Phages, selection and propagation

Bacteriophage K was obtained from Dr. Christiane Wolz (University of Tübingen, Germany). Phage 812 was described previously^1^. Phages designated Stafal, Pyophage, and StaphPhage were isolated from the commercial therapeutic preparations Stafal (Bohemia Pharmaceuticals, Czech Republic), PyoPhage (Eliava Biopreparations, Georgia), and Staphylococal bacteriophage liquid (Microgen, Russia), respectively, and propagated on strain CCM 8428 as described previously^45^. Phages 812 and 812h1 were deposited in the Czech Collection of Microorganisms under accession number CCM 7911 and in the Collection of Animal Pathogenic Microorganisms under accession number CAPM V-689, respectively. Phage 812K1/420 was patented for treating human MRSA infections^46^.

Phage 812 was propagated using stock lysate. After the seeding of the parental phage with a strain resistant to this phage (selection strain) into 0.7% meat-peptone soft agar overlaid on 1.5% meat peptone agar (MPA) plates, individual rare plaques of the mutant phages were picked into 0.1 ml of a liquid medium, meat-peptone broth (MPB), prepared from 13 g of nutrient broth (Oxoid, CM0001), 3 g of yeast extract (Oxoid, LP0021), and 5 g of peptone (Oxoid, LP0037) dissolved in distilled water to a volume of 1000 ml (pH 7.4). Suspensions were purified through a 0.45 µm OlimPeak nylon filter (Teknokroma, Barcelona, Spain) to remove the remaining bacterial cells. Phage mutant 812a was derived from the wild-type phage 812 by selection on *S. aureus* strain NCTC 8511 (ST254, t118) laboratory lysogenized by phage ϕ53^47^. The mutant 812a served as a parental phage to produce the mutants 812K1 and 812F1 isolated on *S. aureus* strains FN34 (ST5, t002) and FN4 (ST1021, t11517), respectively^1^. Phage mutant 812K1/420 was derived from phage 812K1 by selection on human MRSA strain NRL Atb 420 (ST5, t179). Phage 812h1 was selected on MRSA strain 1098 (ST8, t064) from mutant 812h which was selected from phage 812 on MSSA strain 347 (ST5, t002).

Large-scale phage propagation on *S. aureus* strains was performed in 250 ml MPB with aeration. The phages 812, 812a, 812F1, and 812K1 were propagated on strain CCM 4028 (ST30, t021). Phage 812K1/420 was propagated on prophage-less strain CCM 8428 (ST30, t021). Phage 812h1 was propagated on prophage-less MSSA strain CAPM 6630 (ST6, t304). To prove the genetic stability of the obtained phage mutants and to exclude the possibility of phage DNA modification, the phage lytic activity was tested on the selection strain after passaging on the propagation strain, and the efficiency of plating (EOP) was determined. EOP was calculated by dividing the phage titer obtained on selection strains by the phage titer on the propagating strain^1^.

### Phage susceptibility testing

*S. aureus* strains were grown in MPB to the logarithmic phase at 37 °C. Meat peptone agar plates (1,5% agar) with 2 mM CaCl_2_ were flooded with the broth culture, drained, and dried. The phage lysates of titer of 10^9^ plaque forming units (PFU) per ml were diluted 10^−2^ (i.e. 10^7^ PFU/ml) and 10^−4^ (i.e. 10^5^ PFU/ml) and applied in triplicate for both the dilutions by spotting 10 µl aliquots onto soft agar lawns inoculated with tested *S. aureus* strain. Plates were incubated overnight at 37 °C. Then the results were evaluated (Supplementary Fig. S3). If single plaques appeared, the strain was considered susceptible (Supplementary Fig. S3A). If no plaques appeared in any dilution, the strain was considered phage-resistant (Supplementary Fig. S3B).

### Phage genome sequencing and analysis

Phenol/chloroform extraction of DNA was performed according to Sambrook *et al*.^48^. The procedure was modified so that Phase Lock Gel tubes (5 PRIME, Hamburg, Germany) were used for separating the aqueous and organic phases. The complete nucleotide sequences (30 to 50-fold coverage) of bacteriophages 812a, 812K1, 812F1, and 812K1/420 were determined using the GS Junior system (Roche, 454 Life Science, USA) according to a previous study^49^. The genomes of phages 812 and 812h1 were determined using the MiSEQ system (Illumina, USA) according to Botka *et al*.^50^. The reads were assembled using the A5-miseq pipeline^51^. The genomes were covered, on average, 2272-fold in phage 812 and 3076-fold in phage 812h1. Sequence gaps were closed using PCR and primer walking. Amplified PCR products were purified using a QIAquick PCR Purification kit (Qiagen, Netherlands) and sequenced by Eurofins MWG Operon (Germany). The same approach was used for PCR assays to verify extensive DIPs of phage 812h1 and to analyse tandem repeat regions. Primers and polymerase chain reaction conditions for differentiation of the phage 812-derived mutants are described in Supplementary Table S6.

The GenBank (NCBI) accession numbers of the phage genomes are as follows: updated genome of phage 812 (MH844528), and genomes of phages 812a (KJ206560), 812K1 (KJ206561), 812F1 (KJ206562), 812K1/420 (KJ206563), and 812h1 (MH844529).

Variations in the genomes obtained with the MiSEQ system were detected using CLC Genomic Workbench v3.6.5 (Qiagen Bioinformatics, Denmark) by analysing the set of pair-end reads aligned to the genome sequence under the following conditions: coverage of reads ≥90% and identity ≥90%, ignoring non-specific matches. To exclude sequencing errors, only the SNPs and DIPs with at least 10% frequency and, at the same time, a minimum of 30-fold coverage in low-coverage parts were considered. The quality conditions were as follows: quality of central base and average quality of surrounding bases ≥Q30. DNA samples were verified by multiplex PCR assay (Supplementary Table S6) to exclude cross-contamination.

Taking into account that the redundant regions exhibit multiplied coverage compared to the rest of the genomes^35^, the long terminal repeats were predicted based on a significant change in the MiSEQ read number of the alignments. Open reading frames (ORFs) were identified using GeneMarkS, optimized for phage sequences (http://exon.gatech.edu) and inspected manually. Genomes were annotated using RAST^52^, Phobius (http://phobius.sbc.su.se), and BLAST (http://blast.ncbi.nlm.nih.gov/). The EMBOSS tools Needle and Stretcher (https://www.ebi.ac.uk/) were used for pairwise sequence comparisons. The tRNA genes were identified using tRNAscan-SE^53^ and RAST. To predict promoters specific for the host and phage sigma factors, 200-bp sequences upstream of start codons were investigated using GLAM2 v.5.0.2 from MEME Suite^54^ and BPROM^55^, with only the promoters that reached an overall score (LDF) above 5 being considered. Only one promoter per transcription unit was taken into account. ARNold^56^ was applied to Rho-independent terminator identification. Only the predicted terminators with a dG value of less than −7.0 kcal/mol and a functional position were taken into consideration. The genome maps were constructed using GCView^57^. The ARDB^58^ and VFDB^59^ databases were used to search for antibiotic resistance and virulence factor genes. Parameters for ARDB searching were as follows: blastp program, complete database of resistance genes, 40% identity, and an E-value of 0.0001. Parameters for VFDB searching were as follows: tblastn program, using whole phage sequences against DNA sequences from the VFDB datasets A and B. Phylogenetic trees were constructed using the maximum likelihood method (aLRT test) performed in the on-line tool Phylogeny.fr^60^. Genomic sequences of related phages ISP (FR852584), fRuSau02 (MF398190), JD007 (NC_019726), Staph1N (JX080300), A3R (JX080301), 676Z (JX080302), Fi200W (JX080303), P4W (JX080305), K (KF766114), SA012 (NC_023573), G1 (AY954969), IME-SA1 (KP687431), IME-SA2 (KP687432), GH15 (NC_019448), JA1 (MF405094), IPLA-RODI (NC_028765), S25-4 (NC_022918), SA3 (MF001365), SA5 (JX875065), Sb-1 (HQ163896), Team1 (NC_025417), A5W (EU418428), P108 (NC_025426), and qdsa002 (KY779849)^2–4,6,20,23,32,33,34,61–67^ were retrieved from the NCBI Nucleotide database (https://www.ncbi.nlm.nih.gov/nucleotide).

### qPCR quantification of bacterial genes *blaZ, tetK* and *mecA* inside phage particles

Phages K, 812K1/420, PyoPhage and 80α were propagated on MRSA strains COL (pT181) and USA300 08/019 (pUSA-Houmr-like)^68^, and the strain RN4220 with plasmids pT181 and pUSA-Houmr-like prepared in this study. Extraction of phage DNA including DNase treatment was performed as described previously^69^. A LightCycler 480 Instrument II (Roche) was used for qPCR performed according to Mašlaňová *et al*.^69^. Reactions were carried out in triplicates in optical 96-well reaction plates. Each reaction mixture (25 μl) contained 12.5 μl LightCycler 480 SYBR Green I Master (Roche), 300 µM of each primer (Supplementary Table S5) and 25 ng of template DNA in a volume of 2.5 µl. Genes encoding distinctive siphoviral and myoviral structural proteins^44^ were used as a reference.

## Supplementary information

Supplementary material

## Data Availability

All data generated or analysed during this study are included in this published article (and its Supplementary files), or the relevant depository/collection/database is indicated in Materials and Methods. The large datasets (qPCR, raw sequence data) generated during and/or analysed during the current study are available from the corresponding author on request.
