# Supplementary material for: Lytic and genomic properties of spontaneous host-range Kayvirus mutants prove their suitability for upgrading phage therapeutics against staphylococci
Source: Sci Rep. 2019 Apr 2;9:5475. doi: 10.1038/s41598-019-41868-w (PMC6445280; doi:10.1038/s41598-019-41868-w)
Supplement: Supplementary file 1 — Supplementary material [file 41598_2019_41868_MOESM1_ESM.pdf]

SUPPLEMENTARY MATERIAL

Lytic and genomic properties of spontaneous host-range *Kayvirus* mutants prove their suitability for upgrading phage therapeutics against staphylococci

Tibor Botka[1], Roman Pantůček[1*], Ivana Mašlaňová[1], Martin Benešík[1], Petr Petráš[2], Vladislava Růžičková[1], Pavla Havlíčková[1], Marian Varga[1], Helena Žemličková[2,3], Ivana Koláčková[4], Martina Florianová[4], Vladislav Jakubů[2], Renáta Karpíšková[4], Jiří Doškař[1]

[1]Department of Experimental Biology, Faculty of Science, Masaryk University, Brno, 611 37, Czech Republic

[2]National Institute of Public Health, Praha, 100 42, Czech Republic

[3]Department of Clinical Microbiology, University Hospital and Faculty of Medicine in Hradec Králové, Charles University, Hradec Králové, 500 05, Czech Republic

[4]Veterinary Research Institute, Brno, 621 00, Czech Republic

[*]pantucek@mail.muni.cz, phone: +420 549496379

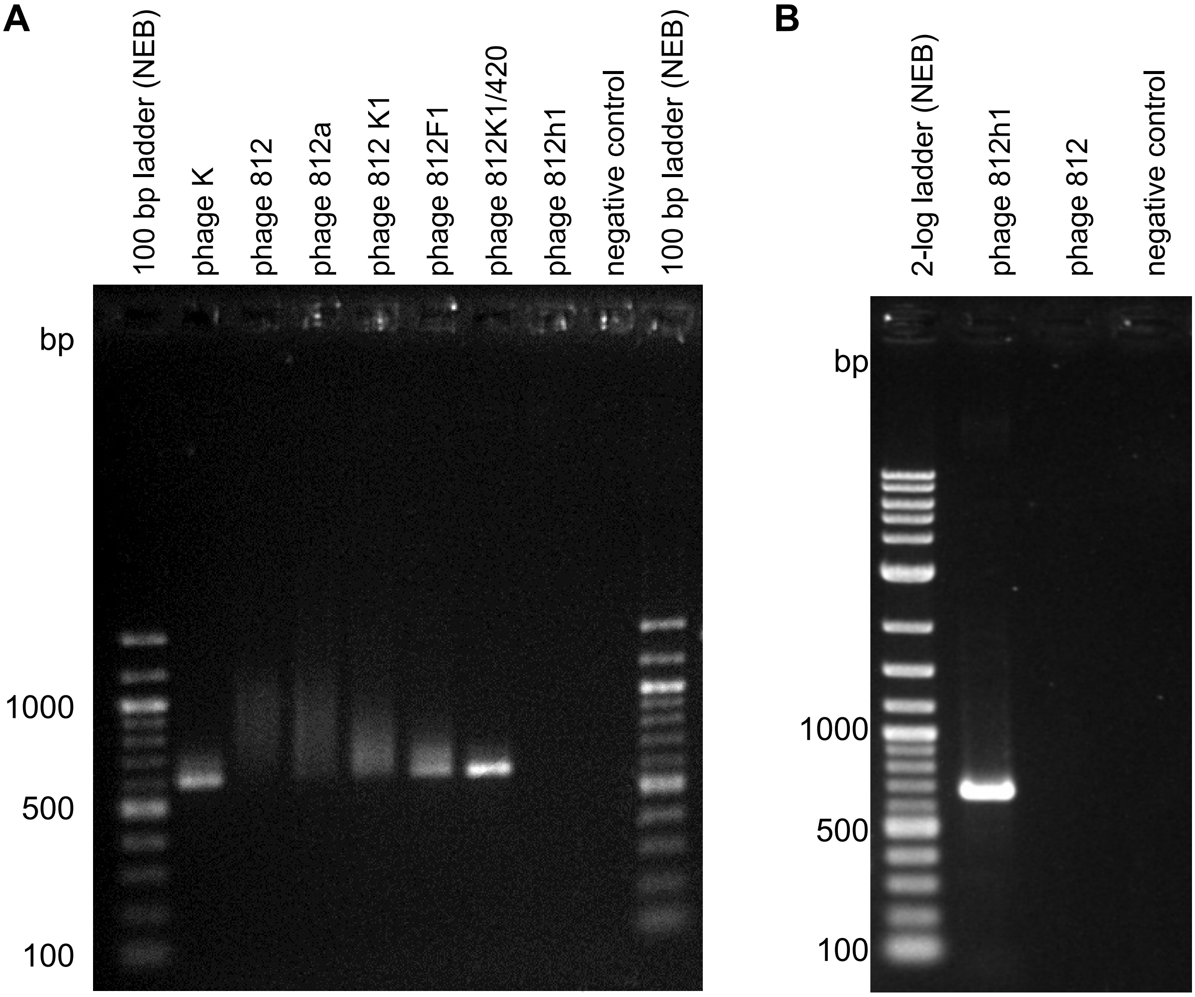

Figure S1. PCR amplification of the region harbouring 35-bp tandem repeat between the unit genome and right long terminal repeat (R-LTR). (A) PCR was performed using primers (5' - 3') TR_F: AGGCAAGGATTGGGATAAACAAG and TR_R: AGTACCACTTTTCCTA-GGGTC to detect the region at the end of the unit genome. In the phage K, the amplified 599-bp sequence is in coordinates 139,484 (unit genome) – 140,082 (R-LTR) and contains two copies of 35-bp tandem repeat. As the figure shows, the phage 812h1 does not contain sequence complementary to the primer TR_F. (B) In this case, the primer TR-812h1_F: TGGTAAGGGAGGGAATTAATATGAT was used together with TR_R; and the amplicon with 677 bp in size was detected. Full-length 1.5% agarose gels are presented.

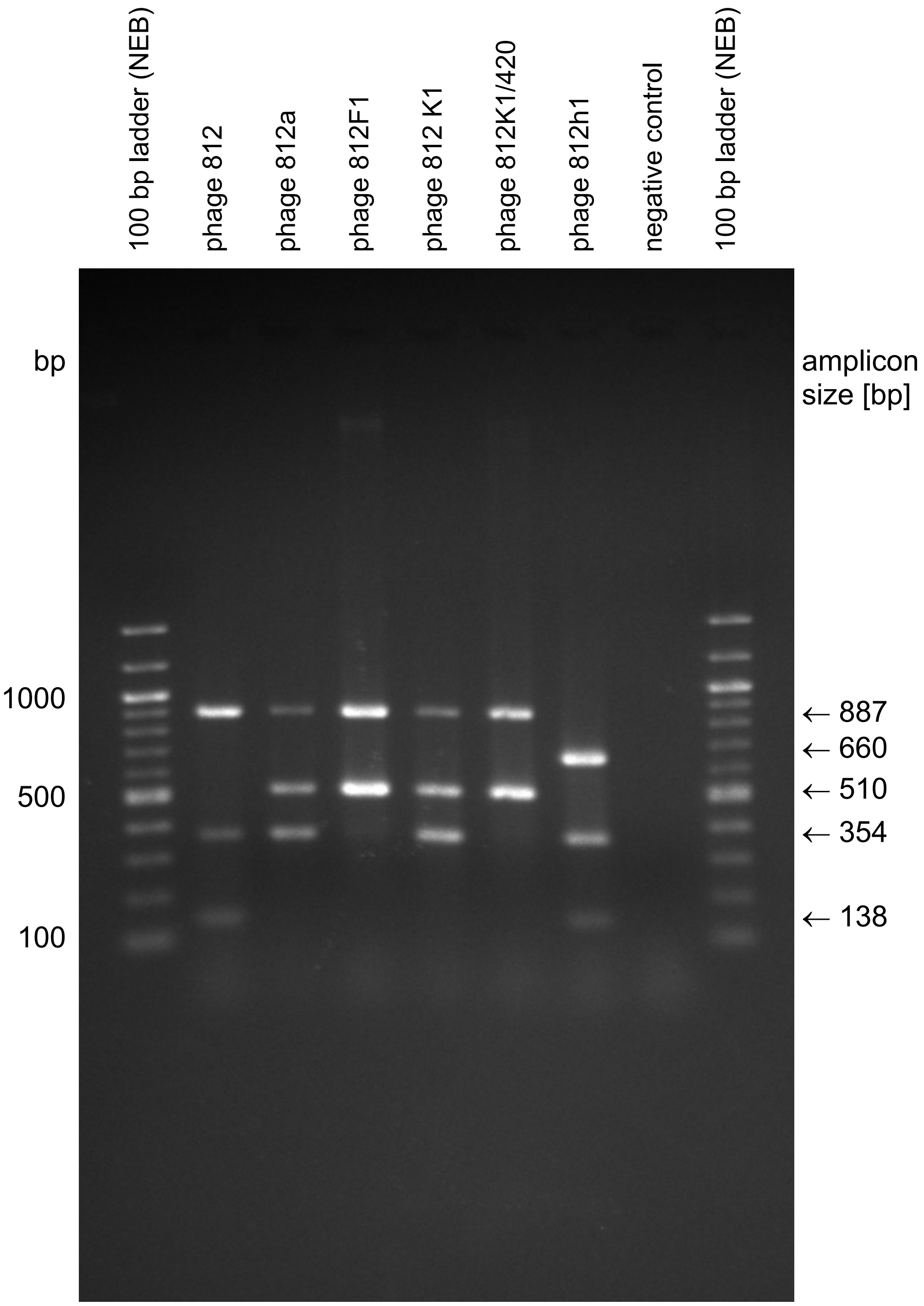

Figure S2. PCR assay for differenciation of phage 812-derived mutants. Due to high similarity, the phages 812a and 812K1, and 812F1 and 812K1/420 exhibit identical amplicon profiles. Primers and polymerase chain reaction conditions are described in Supplementary Table S6. OneTaq Quick-Load 2X Master Mix with Standard Buffer (New England Biolabs, USA) was used to perform PCR. Full-length 1.5% agarose gel is presented.

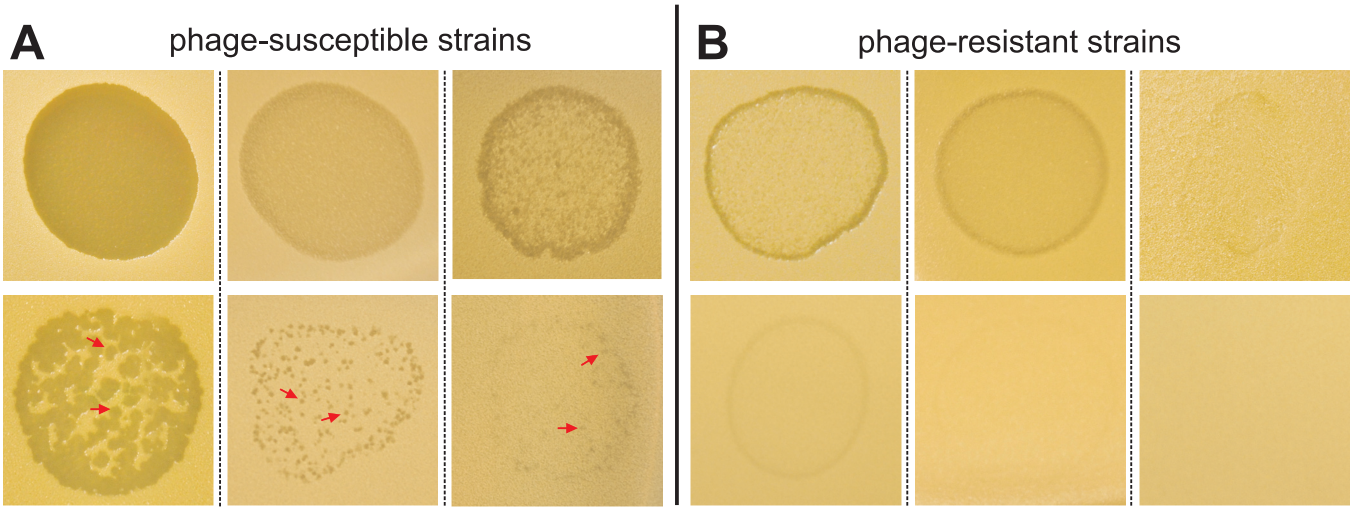

Figure S3. Different types of *Kayvirus* phage susceptibility test results demonstrated on three susceptible and three resistant *S. aureus* strains. High-titer phage stock lysates of 10^9 plaque forming units per ml (PFU/ml) were diluted for the testing and applied by spotting 10 µl aliquots onto soft agar lawns inoculated with tested *S. aureus* strain. The observed lytic reactions with lysates diluted 10^{-2} (i.e. 10^7 PFU/ml) are shown in the top line and the lytic reactions with lysates diluted 10^{-4} (i.e. 10^5 PFU/ml) are shown in the bottom line.

(**A**) Bacterial strain was considered phage-susceptible if single plaques (examples are marked by red arrows) appeared.

(**B**) Bacterial strain was considered phage-resistant if no plaques appeared.

Table S1. Phage susceptibility of MRSA strains. The strains are grouped according their genotype (clonal complex - CC, sequence type - ST, and *spa* type). Phage-resistant (-) or phage-susceptible (+) phenotype of each group as well as the total number of resistant and susceptible strains is given to each phage studied.

[1] CC1, CC5, and CC8 are recently all connected by eBURST v3.

MRSA strains						Phage susceptibility									
CC[1]	MLST	*spa*	origin	no. of isolates		K	812	812a	812F1	812K1	812K1/420	812h1	Stafal	PyoPhage	StaphPhage
CC1	ST1	t127	environment	1		-	-	-	-	-	-	-	-	-	-
CC1	ST1	t127	human	1		-	-	-	-	-	-	+	+	+	-
CC1	ST1	t1909	environment	1		+	-	+	-	-	-	-	-	-	-
CC5	ST5	t002	human	2		-	-	-	-	-	-	+	-	+	+
CC5	ST5	t002	human	1		-	+	-	-	-	-	+	+	-	-
CC5	ST5	t002	human	1		-	+	-	-	-	-	+	+	+	+
CC5	ST5	t002	human	1		+	+	+	+	+	+	+	+	-	-
CC5	ST5	t002	human	2		+	+	+	+	+	+	+	+	+	+
CC5	ST5	t010	human	2		+	+	+	+	+	+	+	+	+	+
CC5	ST5	t105	human	1		+	+	+	+	+	+	-	+	+	-
CC5	ST5	t179	human	1		-	-	-	+	-	+	-	-	-	-
CC5	ST5	t179	human	2		+	+	-	+	+	+	+	+	-	-
CC5	ST5	t311	human	2		-	-	-	-	-	-	+	-	-	-
CC5	ST5	t311	human	1		+	+	+	+	+	+	+	+	+	+
CC5	ST111	t041	human	1		-	+	+	-	+	-	+	+	+	+
CC5	ST111	t041	human	1		+	+	+	+	+	+	+	+	+	+
CC5	ST111	t3193	human	1		-	+	-	-	-	-	-	+	-	-
CC5	ST225	t003	human	1		-	-	-	+	-	+	-	-	-	-
CC5	ST225	t003	human	7		+	+	+	+	+	+	+	+	+	+
CC5	ST225	t014	human	1		+	+	+	+	+	+	+	+	+	+
CC5	ST225	t045	human	1		-	+	-	-	-	-	+	-	+	+
CC5	ST225	t521	human	1		+	+	+	+	+	+	+	+	+	+
CC5	ST225	t3195	human	1		+	+	+	+	+	+	+	+	+	+
CC5	ST228	t041	human	1		+	+	+	+	+	+	+	+	+	+
CC8	ST8	t008	human	3		-	-	-	-	-	-	+	-	-	-
CC8	ST8	t008	human	1		-	-	-	-	-	-	+	-	+	+
CC8	ST8	t008	human	1		-	-	+	+	+	+	+	-	+	+
CC8	ST8	t008	human	1		+	+	-	-	-	-	+	+	+	+
CC8	ST8	t008	human	11		+	+	+	+	+	+	+	+	+	+
CC8	ST8	t024	human	1		-	-	-	-	-	-	-	-	-	-
CC8	ST8	t024	human	5		+	+	+	+	+	+	+	+	+	+
CC8	ST8	t024	environment	1		-	-	-	-	-	-	+	-	+	-
CC8	ST8	t064	livestock	4		-	-	-	-	-	-	+	-	+	+
CC8	ST8	t088	human	1		-	-	-	+	-	+	-	-	+	-
CC8	ST8	t121	human	1		+	+	+	+	+	+	+	+	-	+
CC8	ST8	t197	human	1		-	-	-	+	-	+	-	-	-	-
CC8	ST8	t711	human	1		+	+	+	+	+	+	+	+	+	+
CC8	ST239	t030	human	3		-	-	-	-	-	-	+	-	+	+
CC8	ST239	t030	human	1		+	+	+	+	+	+	+	+	+	+
CC8	ST239	t037	human	2		-	-	-	-	-	-	+	-	+	+
CC8	ST239	t037	human	1		+	+	+	+	+	+	+	+	+	+
CC8	ST239	t3167	human	1		-	-	-	-	-	-	+	-	+	+
CC8	ST247	t051	human	1		+	+	+	+	+	+	+	+	+	+
CC8	ST247	t211	human	1		-	+	-	-	-	-	+	+	+	+
CC8	ST247	t211	human	1		+	+	+	+	+	+	+	+	+	+
CC8	ST624	t211	human	1		-	+	-	-	-	-	-	+	+	+
CC20	ST20	t195	human	1		-	+	-	-	-	-	+	+	+	+
CC22	ST22	t020	human	1		-	-	-	+	-	+	-	-	-	-
CC22	ST22	t032	human	1		-	-	-	-	-	-	-	-	-	-
CC22	ST22	t032	human	1		-	-	+	+	+	+	+	-	+	+
CC22	ST22	t032	human	1		+	+	+	+	+	+	+	+	-	-
CC22	ST22	t032	human	2		+	+	+	+	+	+	+	+	+	+
CC22	ST22	t223	human	1		-	-	-	-	-	+	-	-	+	+
CC30	ST30	t019	human	1		+	+	+	+	+	+	+	+	+	+
CC45	ST45	t015	human	1		-	-	-	-	-	-	-	-	-	-
CC45	ST45	t015	environment	1		-	-	-	-	-	-	-	-	-	-
CC45	ST45	t015	human	1		-	-	-	+	-	+	-	-	-	-
CC45	ST45	t026	human	1		-	-	-	-	-	-	-	-	-	-
CC45	ST45	t026	human	1		-	-	+	+	+	+	+	-	+	+
CC45	ST45	t065	human	1		-	-	-	-	-	-	-	-	-	-
CC45	ST45	t230	human	1		-	-	-	-	-	-	-	-	-	-
CC80	ST80	t044	human	1		-	-	-	-	-	-	-	-	-	-
CC80	ST80	t044	human	4		+	+	+	+	+	+	+	+	+	+
CC88	ST88	t186	human	1		-	-	-	-	-	-	+	+	-	-
CC96	ST154	t667	human	1		-	-	-	-	-	-	+	-	+	+
CC398	ST398	t011	livestock	1		-	-	-	-	-	-	+	-	+	+
CC398	ST398	t011	livestock	1		+	-	-	-	-	-	+	+	+	-
CC398	ST398	t011	livestock	1		-	-	-	-	-	+	+	-	+	+
CC398	ST398	t011	livestock	1		-	-	-	+	-	+	+	+	+	+
CC398	ST398	t011	livestock	1		+	-	-	+	+	-	+	+	+	+
CC398	ST398	t011	livestock	3		+	-	-	-	-	+	+	+	+	+
CC398	ST398	t011	livestock	1		-	-	+	+	+	+	+	+	+	+
CC398	ST398	t011	livestock	3		+	-	+	+	+	+	+	+	+	+
CC398	ST398	t011	livestock	30		+	+	+	+	+	+	+	+	+	+
CC398	ST398	t034	livestock	1		-	-	-	+	-	+	+	-	+	+
CC398	ST398	t034	livestock	1		-	-	+	+	+	+	+	-	+	+
CC398	ST398	t034	livestock	1		+	-	+	+	+	+	+	+	+	+
CC398	ST398	t034	livestock	23		+	+	+	+	+	+	+	+	+	+
CC398	ST398	t899	livestock	1		+	+	+	+	+	+	+	+	+	+
CC398	ST398	t1255	livestock	1		+	-	-	+	+	+	+	+	+	+
CC398	ST398	t2346	livestock	1		-	-	-	+	-	+	+	+	+	+
CC398	ST398	t2346	livestock	3		+	+	+	+	+	+	+	+	+	+
CC398	ST398	t4652	livestock	1		+	+	+	+	+	+	+	+	+	+
CC398	ST398	t4659	livestock	1		+	+	+	+	+	+	+	+	+	+
CC672	ST361	t011	livestock	1		-	-	-	+	+	-	+	-	-	-
CC672	ST361	t315	livestock	6		+	+	+	+	+	+	+	+	+	+
CC672	ST361	t315	livestock	1		+	-	+	+	+	+	+	+	+	+
Total				186	-	57	61	60	48	56	45	20	46	29	33
					+	129	125	126	138	130	141	166	140	157	153

Table S2. Polymorphisms detected in phage 812 genome.

Type	Position	ORF	Product	Frequency	nt change	Effect
SNP	16198	37	hypothetical protein	86.3/13.7	G/A	His -> Tyr
SNP	16944	40	transglycosylase	88.2/11.8	G/A	-
SNP	23723	55		88.2/11.8	G/A	-
SNP	23767	55		81.5/18.5	G/C	Gln -> Glu
SNP	23771	55	hypothetical protein	81.1/18.8	C/T	-
SNP	23783	55		79.7/20.3	A/T	-
SNP	23806	55		79.9/20.1	C/T	Asp -> Asn
SNP	24817	55		60.4/39.6	A/C	Tyr -> Asp
SNP	25730	58	hypothetical protein	55.8/44.2	C/T	-
DIP	46177^46178	89	hypothetical protein	89.7/10.3	------/CCTGAA	no frameshift
SNP	56366	102		63.2/36.8	C/T	Thr -> Ile
SNP	56461	102		63.4/36.6	G/T	Ala -> Ser
SNP	57428	102	tail sheath protein	81.7/18.3	T/G	Val -> Gly
SNP	57824	102		83.2/16.8	G/A	Arg -> Gln
SNP	58001	102		84.3/15.7	T/G	Ile -> Ser
SNP	60675	105		85.5/14.5	C/T	-
SNP	60678	105		85.4/14.6	A/G	-
SNP	60693	105	hypothetical protein	81.5/18.5	T/A	-
SNP	60699	105		83.2/16.8	A/G	Ile -> Met
SNP	60706	105		78.6/21.3	T/A	Ser -> Thr
SNP	62616	110		85.7/14.3	C/T	-
SNP	62640	110		51.0/49.0	C/T	-
SNP	62676	110		66.3/33.7	C/T	-
SNP	62697	110		64.4/35.6	C/T	-
SNP	62836	110	tail morphogenetic protein	64.5/35.5	G/A	Val -> Met
SNP	62892	110		62.9/37.1	G/A	-
SNP	63009	110		64.9/35.1	G/T	Glu -> Asp
SNP	63012	110		64.2/35.8	T/C	-
SNP	63148	111		70.3/29.7	T/C	-
SNP	63199	111		71.1/28.9	G/A	-
SNP	63568	111		72.2/27.7	T/C	-
SNP	63733	111		57.1/42.8	A/T	-
SNP	63736	111		57.4/42.6	A/G	-
SNP	63769	111		67.9/31.9	G/A	-
SNP	63779	111		67.8/32.2	A/G	Ile -> Val
SNP	63823	111		68.5/31.5	A/G	-
SNP	63825	111		67.4/32.6	G/A	Gly -> Glu
SNP	63838	111		68.4/31.6	G/A	-
SNP	63859	111		67.3/32.7	T/C	-
SNP	63909	111		67.3/32.7	A/C	Glu -> Ala
SNP	64057	111		66.6/33.4	T/C	-
SNP	64070	111		66.7/33.3	C/A	-
SNP	64178	111		74.1/25.9	C/T	-
SNP	64198	111		73.7/26.3	T/G	-
SNP	64279	111		73.3/26.7	T/C	-
SNP	64282	111		72.8/27.2	C/T	-
SNP	64312	111		71.7/28.3	G/A	-
SNP	64411	111	tape measure protein	90.0/10.0	C/T	-
SNP	64416	111		89.8/10.2	A/G	Lys -> Arg
SNP	64462	111		89.1/10.9	C/T	-
SNP	64627	111		87.6/12.4	C/T	-
SNP	64774	111		87.6/12.4	G/A	-
SNP	64852	111		87.2/12.8	A/G	-
SNP	64867	111		87.5/12.5	A/G	Ile -> Met

Table S2. Continued.

SNP	64942	111		87.5/12.5	C/T	-
SNP	64975	111		71.8/28.2	A/G	-
SNP	64978	111		72.3/27.7	T/C	-
SNP	64979	111		72.5/27.5	G/T	Ala -> Ser
SNP	64982	111		71.9/28.1	C/A	Gln -> Lys
SNP	64985	111		71.7/28.3	T/G	Ser -> Ala
SNP	64986	111		72.2/27.8	C/A	Ser -> Tyr
SNP	64987	111		72.3/27.7	C/A	-
SNP	64996	111		86.4/13.6	G/A	-
SNP	65116	111		87.8/12.2	T/G	-
SNP	65218	111		88.3/11.6	T/A	-
SNP	65221	111		88.4/11.6	T/A	-
SNP	68214	112	tail murein hydrolase	59.8/40.2	A/C	His -> Pro
SNP	68910	112		60.7/39.1	C/G	Ala -> Gly
SNP	69859	113	cysteine protease	62.1/37.9	C/T	Thr -> Ile
SNP	71627	114	tail central spike	56.8/43.2	G/A	Glu -> Lys
SNP	72173	114		72.9/27.1	T/G	Leu -> Val
SNP	74087	116	baseplate component	55.1/44.8	G/A	Thr -> Ala
SNP	76788	119	tail fibre protein complex	84.8/15.2	A/C	Asn -> Thr
SNP	78732	119		71.9/28.1	A/C	Tyr -> Ser
SNP	82389	121	adsorption-associated tail protein	86.8/13.2	T/G	Ser -> Ala
SNP	84927	123	receptor binding protein	80.8/19.2	T/G	Phe -> Val
SNP	85149	123		80.6/19.4	G/T	Gly -> Cys
SNP	85294	123		76.2/23.8	G/C	Ser -> Thr
SNP	85554	123		60.8/39.2	T/A	Thr -> Ser
SNP	86175	125	receptor binding protein	73.0/27.0	A/C	Leu -> Phe
SNP	87200	125		62.3/37.7	A/C	Asn -> Thr
SNP	87292	125		89.8/10.1	G/A	Asp -> Asn
SNP	92345	-	-	63.8/36.2	A/C	-
SNP	97894	135	hypothetical protein	68.8/31.2	T/C	Ala -> Val
SNP	97950	135		69.0/31.0	T/G	Tyr -> Asp
SNP	99498	138	ribonucleotide reductase large subunit	60.2/39.8	G/C	Lys -> Asn
SNP	99768	138		75.6/24.4	C/T	-
SNP	99774	138		75.4/24.6	A/G	-
SNP	99858	138		72.9/27.1	T/C	-
SNP	99885	138		72.7/27.3	T/C	-
SNP	99975	138		75.6/24.4	T/C	-
SNP	100044	138		76.0/24.0	C/T	-
SNP	100059	138		76.8/23.2	C/T	-
SNP	100069	138		75.9/24.1	T/C	-
SNP	100083	138		77.2/22.8	C/T	-
SNP	100101	138		77.1/22.9	C/T	-
SNP	100119	138		76.9/23.0	A/C	-
SNP	100219	138		52.9/47.1	G/A	Met -> Val
SNP	100276	138		56.5/43.5	G/A	Asp -> Asn
SNP	100329	138		57.3/42.6	T/C	-
SNP	100341	138		56.6/43.3	G/A	-
SNP	100356	138		57.3/42.7	C/G	-
SNP	100485	138		79.1/20.9	T/C	-
SNP	100524	138		79.0/21.0	T/C	-
SNP	100527	138		78.7/21.3	C/T	-
SNP	100557	138		78.2/21.8	A/G	-
SNP	101764	139		68.0/32.0	G/T	-
SNP	101776	139		67.5/32.5	C/T	-
SNP	101809	139		67.4/32.6	T/C	-

Table S2. Continued.

SNP	101813	139	ribonucleotide reductase small subunit	68.3/31.7	T/C	-
SNP	101830	139		68.9/31.1	A/T	-
SNP	101869	139		69.3/30.7	C/T	-
SNP	101881	139		69.9/30.1	C/T	-
SNP	101891	139		68.6/31.4	T/C	-
SNP	101902	139		67.6/32.4	T/C	-
SNP	102064	139		71.8/28.2	T/C	-
SNP	102209	139		69.2/30.8	C/T	-
SNP	102283	139		68.1/31.9	G/A	-
SNP	102310	139		68.7/31.3	C/T	-
SNP	102609	140	hypothetical protein	51.9/48.1	G/A	-
SNP	102636	140		50.7/49.3	T/G	Lys -> Asn
SNP	102700	140		50.1/49.9	T/C	-
SNP	102748	140		51.9/48.1	A/G	Ile -> Val
SNP	103053	141	oxidoreductase	51.1/48.9	G/A	Gly -> Asp
SNP	103061	141		51.2/48.8	C/T	-
SNP	106645	144	DNA polymerase	56.7/43.3	T/C	Tyr -> His
SNP	114795	154	tail protein	42.3/57.7	C/A	Tyr -> stop codon
SNP	115115	154		54.1/45.9	T/C	Val -> Ala
SNP	120362	165	hypothetical protein	55.5/44.5	C/T	-
SNP	122654	167	putative structural protein	75.7/24.3	C/T	Thr -> Ile
SNP	127566	181	hypothetical protein	81.1/18.9	T/G	Ile -> Ser
SNP	128637	184	hypothetical protein	85.0/15.0	A/G	Ser -> Gly
SNP	131288	-	-	89.7/10.3	T/A	-
DIP	138249^138250	208	nicotinate phosphoribosyltransferase	82.1/17.9	-/A	frameshift
SNP	138252	208		80.5/19.5	C/T	Thr -> Ile
DIP	138256	208		80.4/19.6	A/-	frameshift
SNP	138259	208		79.7/20.3	T/C	-
SNP	138262	208		80.0/20.0	A/G	-
SNP	138266	208		77.1/22.9	C/T	-
SNP	138271	208		70.3/29.7	C/T	-
SNP	138281	208		71.9/28.1	G/T	Val -> Phe
SNP	138283	208		71.9/28.1	T/A	-
SNP	138286	208		70.7/29.3	T/C	-
SNP	138296	208		69.9/30.1	A/C	-
SNP	138319	208		62.3/37.7	T/A	-
SNP	138323	208		62.0/38.0	A/C	-
SNP	138487	209	hypothetical protein	75.0/25.0	G/A	-
SNP	138493	209		81.5/18.5	A/G	-
SNP	138494	209		81.8/18.2	T/C	Tyr -> His
SNP	138499	209		85.0/15.0	A/C	-
SNP	138500	209		84.3/15.7	T/A	Ser -> Thr
SNP	138506	209		85.2/14.8	T/C	-
SNP	138507	209		85.2/14.8	T/C	Leu -> Ser
SNP	138508	209		84.6/15.4	A/T	Leu -> Phe
SNP	138517	209		87.4/12.6	A/G	-
SNP	138520	209		87.5/12.5	A/G	-
SNP	138704	-	-	79.6/20.4	G/A	-
SNP	138705	-	-	79.5/20.5	T/A	-
SNP	138709	-	-	74.2/25.8	C/A	-
SNP	138746	210		55.0/45.0	G/A	-
SNP	138749	210		53.9/46.1	T/A	-
SNP	138752	210		55.2/44.8	C/T	-
SNP	138755	210		51.4/48.6	G/T	-
SNP	138761	210		53.7/46.3	C/T	-

Table S2. Continued.

SNP	138933	210		51.0/49.0	T/C	-
SNP	138941	210		54.0/46.0	C/T	-
SNP	138945	210	hypothetical protein	55.4/44.6	A/G	Asn -> Asp
SNP	138953	210		57.9/42.1	A/G	-
SNP	138959	210		61.4/38.6	A/T	Glu -> Asp
SNP	138977	210		72.3/27.7	A/G	-
DIP	138980	210		73.3/16.4/10.4	G/A/-	frameshift
SNP	138983	210		82.1/17.9	A/G	-
SNP	138986	210		81.6/18.4	G/T	-
SNP	138989	210		81.7/18.3	A/T	-
SNP	138992	210		82.0/18.0	G/A	-
DIP	141889-141926	LTR	-	deletion[1]		-

[1]polymorphism proved by sequencing of correspondent PCR amplicon

Table S3. Polymorphisms detected in phage 812h1 genome.

Type	Position	ORF	Product	Frequency	nt change	Effect
SNP	2444	7	terminal repeat-encoded protein	80.3/19.7	G/T	Arg -> Ile
DIP	2533^2534	7			gene duplication[1]	
SNP	2544	-	-	89.4/10.6	G/A	-
SNP	3786	10		86.4/13.6	A/T	-
SNP	3792	10		86.1/13.9	G/A	-
SNP	3793	10	terminal repeat-encoded protein	86.3/13.7	T/C	-
SNP	3795	10		86.5/13.5	G/A	-
SNP	3820	10		85.3/14.7	G/A	Ala -> Thr
SNP	4132	-		83.4/16.6	C/T	-
SNP	4196	-		84.3/15.7	A/G	-
SNP	4197	-		83.8/16.2	G/T	-
SNP	4201	-	-	84.4/15.5	A/G	-
SNP	4202	-		83.8/16.0	C/A	-
SNP	4204	-		84.6/15.4	A/G	-
SNP	4205	-		84.7/15.2	A/T	-
SNP	4219	-		83.1/16.9	C/A	-
SNP	7674	19	terminal repeat-encoded protein	88.6/11.3	G/A	-
SNP	7705	19		87.2/12.8	G/A	Val -> Ile
SNP	7744	20		85.3/14.7	A/G	Asn -> Ser
SNP	7760	20		84.0/15.9	C/T	-
SNP	7778	20		82.8/17.1	T/A	-
SNP	7799	20		87.0/13.0	G/A	-
SNP	7829	20		87.2/12.7	G/T	Glu -> Asp
SNP	7830	20	terminal repeat-encoded protein	87.3/12.7	C/G	Gln -> Glu
SNP	7832	20		87.4/12.5	G/C	Gln -> His
SNP	7840	20		83.0/17.0	A/C	Asn -> Thr
SNP	7841	20		82.6/17.4	T/A	Asn -> Lys
SNP	7844	20		83.2/16.7	T/G	Asp -> Glu
SNP	7866	20		81.8/18.2	A/G	Arg -> Gly
SNP	7871	20		81.3/18.7	C/T	-
SNP	8452	-	-	84.3/15.7	A/G	-
SNP	8729	22	hypothetical protein	80.1/19.9	T/A	-
SNP	9947	25	hypothetical protein	83.0/17.0	C/T	-
SNP	9961	26		83.2/16.8	A/C	stop codon-> Glu
SNP	10036	26		79.7/20.3	A/G	-
SNP	10049	26		79.6/20.4	G/A	-
SNP	10052	26	hypothetical protein	80.8/19.2	T/A	-
SNP	10081	26		79.6/20.4	C/A	Ala -> Ser
SNP	10092	26		79.3/20.7	T/C	Lys -> Arg
SNP	10133	26		80.2/19.8	T/C	-
SNP	10156	26		77.9/22.1	C/T	Ala -> Thr
SNP	10228	27		78.3/21.7	T/C	-
SNP	10242	27	hypothetical protein	79.0/21.0	T/G	-
SNP	10258	27		77.1/22.9	C/T	-
SNP	10901	29	hypothetical protein	74.0/26.0	C/T	Arg -> Lys
SNP	15591	36		69.8/30.1	T/C	Asn -> Asp
SNP	15592	36		69.0/31.0	G/A	-
SNP	15822	36		70.9/29.1	G/T	Leu -> Ile
SNP	15832	36	hypothetical protein	70.7/29.3	C/T	-
SNP	15841	36		71.6/28.4	A/G	-
SNP	15886	36		71.2/28.8	G/A	-
SNP	15894	36		71.9/28.1	A/G	-
SNP	15895	36		71.9/28.1	A/G	-

Table S3. Continued.

SNP	19244	45		75.7/24.3	T/C	-
SNP	19292	45	hypothetical protein	75.8/24.2	A/G	-
SNP	19331	45		76.8/23.2	A/G	-
SNP	19516	46		71.7/28.3	G/A	-
SNP	19552	46		72.6/27.4	T/C	-
SNP	19606	46		73.7/26.3	G/A	-
SNP	19660	46		74.5/25.5	T/C	-
SNP	19672	46	hypothetical protein	75.6/24.4	A/G	-
SNP	19678	46		75.1/24.9	A/G	-
SNP	19780	46		75.3/24.7	C/T	-
SNP	19854	46		75.1/24.9	G/T	-
SNP	20014	47		73.2/26.8	A/G	Val -> Ala
SNP	20016	47	hypothetical protein	72.3/27.7	C/T	Met -> Ile
SNP	20035	47		73.1/26.9	A/G	Leu -> Pro
SNP	20191	48		72.9/27.1	C/T	Asp -> Asn
SNP	20219	48		74.4/25.5	C/T	-
DIP	20223^20224	48	hypothetical protein	78.2/21.5	---/TAT	no frameshift
SNP	20227	48		76.8/23.2	T/C	Ser -> Gly
SNP	20287	48		72.3/27.7	C/T	Val -> Ile
SNP	20294	48		71.4/28.6	C/T	-
SNP	20392	49		74.6/25.4	T/C	-
SNP	20619	49		73.5/26.5	C/T	Val -> Ile
SNP	20647	49		75.1/24.9	T/C	-
SNP	20662	49	hypothetical protein	74.1/25.9	C/T	-
SNP	20737	49		75.0/25.0	G/A	-
SNP	20848	49		72.9/27.1	C/T	-
SNP	20881	49		73.2/26.8	C/T	-
SNP	21504	50		73.5/26.5	A/T	-
SNP	21813	50		75.4/24.6	A/G	-
SNP	21867	50	ATPase	73.6/26.4	C/A	-
SNP	21882	50		73.8/26.2	A/G	-
SNP	21888	50		72.9/27.1	G/A	-
SNP	21903	50		72.5/27.5	G/A	-
SNP	24053	56		70.9/29.1	G/A	-
SNP	25609	56		66.7/33.3	G/T	His -> Asn
SNP	25612	56	hypothetical protein	67.5/32.5	C/T	Gly -> Arg
SNP	25667	56		68.2/31.8	C/T	-
SNP	25693	56		70.3/29.6	C/A	Asp -> Tyr
SNP	25991	56		68.5/31.5	C/T	-
SNP	26605	59	hypothetical protein	69.2/30.8	T/C	Arg -> Gly
SNP	30630	65	ribonuclease H	71.1/28.9	G/T	Ala -> Asp
SNP	40083	-	-	73.5/26.5	C/T	-
SNP	40084	-	-	74.5/25.5	T/A	-
SNP	42491	85	hypothetical protein	73.1/26.9	C/T	-
SNP	43150	86		75.9/24.1	T/C	-
SNP	43303	86		76.9/23.1	C/T	-
SNP	43324	86		76.0/24.0	T/C	-
SNP	43379	86		74.1/25.9	C/T	-
SNP	43408	86		74.5/25.5	A/G	-
SNP	43426	86		75.5/24.5	T/C	-
SNP	43441	86		75.1/24.9	A/T	-
SNP	43624	86		77.2/22.8	A/G	-
SNP	43630	86		77.8/22.2	T/C	-
SNP	43667	86		76.3/23.6	T/C	-

Table S3. Continued.

SNP	43669	86		77.2/22.8	G/A	-
SNP	43684	86		77.1/22.9	C/T	-
SNP	43741	86	terminase large subunit	76.5/23.5	G/A	-
SNP	43744	86		75.7/24.3	T/C	-
SNP	43787	86		76.3/23.7	C/T	-
SNP	43816	86		75.1/24.9	G/T	-
SNP	43852	86		75.8/24.2	G/T	-
SNP	43903	86		74.5/25.5	G/A	-
SNP	44017	86		70.9/29.1	C/T	-
SNP	44026	86		71.6/28.4	T/C	-
SNP	44212	86		71.9/28.1	C/T	-
SNP	44236	86		74.1/25.9	A/C	-
SNP	44239	86		72.2/27.7	G/A	-
SNP	44272	86		74.9/25.1	C/T	-
SNP	44311	86		73.3/26.6	C/T	-
SNP	44392	86		75.3/24.7	A/G	-
SNP	44746	87	putative virion protein	75.2/24.8	T/C	Ile -> Thr
SNP	45020	87		77.1/22.9	G/T	-
SNP	48357	93		76.9/23.1	G/A	-
SNP	48379	93		77.9/22.1	G/A	Val -> Ile
SNP	48525	93	portal protein	74.5/25.5	C/T	-
SNP	48543	93		77.7/22.3	A/G	-
SNP	48555	93		75.8/24.1	T/C	-
SNP	48648	93		74.5/25.5	C/T	-
SNP	54315	98		75.1/24.9	T/C	-
SNP	54330	98	neck protein	74.4/25.6	A/G	-
SNP	54384	98		75.2/24.8	C/T	-
SNP	54604	99		71.4/28.6	C/T	-
SNP	54811	99		73.7/26.3	C/A	-
SNP	54817	99		75.6/24.4	T/C	-
SNP	54838	99		74.7/25.3	G/T	-
SNP	55009	99		78.0/22.0	T/C	-
SNP	55042	99		76.2/23.7	G/A	-
SNP	55061	99	neck protein	77.6/22.4	C/T	-
SNP	55082	99		75.4/24.6	G/A	Val -> Ile
SNP	55105	99		76.3/23.7	T/C	-
SNP	55135	99		75.9/24.1	T/C	-
SNP	55150	99		76.9/23.1	A/G	-
SNP	55246	99		74.9/25.0	C/T	-
SNP	55324	99		75.9/24.1	T/C	-
SNP	55467	100	hypothetical protein	74.3/25.7	C/T	-
SNP	57248	103	tail sheath protein	79.4/20.6	C/T	Thr -> Ile
SNP	62769	109	hypothetical protein	72.1/27.9	C/T	-
SNP	63498	111	tail morphogenetic protein	76.5/23.5	T/C	-
SNP	64450	112		72.2/27.8	C/T	-
SNP	65060	112		73.6/26.4	T/C	-
SNP	65161	112	tape measure protein	72.7/27.3	C/T	-
SNP	65164	112		72.8/27.2	T/C	-
SNP	65194	112		72.9/27.1	A/G	-
SNP	65998	112		72.7/27.3	T/G	-
SNP	72146	115	tail central spike	73.2/26.8	G/A	Gly -> Ser
SNP	85077	124		50.0/49.9	A/G	Asp -> Asn
SNP	85525	124	receptor binding protein	55.4/44.6	A/C	His -> Pro
SNP	85936	124		55.4/44.6	A/C	His -> Pro

Table S3. Continued.

SNP	88174	126	receptor binding protein	54.8/45.2	A/G	Asn -> Asp
SNP	97581	134		65.8/34.2	G/A	-
SNP	97586	134		66.3/33.7	C/A	Thr -> Asn
SNP	97588	134		65.9/34.1	G/A	Ala -> Thr
SNP	97620	134		67.9/32.1	A/G	-
SNP	97665	134		67.2/32.7	T/C	-
SNP	97686	134		66.2/33.8	C/T	-
SNP	97710	134		68.9/31.0	T/C	-
SNP	97719	134		68.3/31.7	T/C	-
SNP	97824	134		67.3/32.7	T/C	-
SNP	97833	134		64.4/35.6	T/C	-
SNP	97843	134		66.1/33.9	C/A	-
SNP	97854	134	DNA primase	67.6/32.4	A/G	-
SNP	97863	134		68.8/31.2	G/T	-
SNP	97878	134		65.9/34.1	C/T	-
SNP	97908	134		66.9/33.1	T/G	-
SNP	98007	134		65.3/34.7	C/A	-
SNP	98034	134		67.1/32.9	T/C	-
SNP	98110	134		65.7/34.3	T/C	-
SNP	98175	134		67.9/32.1	A/T	-
SNP	98181	134		65.7/34.3	G/T	-
SNP	98205	134		68.2/31.8	A/G	-
SNP	98217	134		67.1/32.9	T/	-
SNP	98245	134		66.5/33.5	C/T	-
SNP	98565	135	hypothetical protein	66.8/33.2	C/T	-
SNP	98592	135		69.0/31.0	T/C	-
SNP	98832	136		68.0/32.0	G/T	Asp -> Tyr
SNP	98849	136	hypothetical protein	68.2/31.8	A/T	-
SNP	99005	136		67.4/32.6	T/G	-
SNP	100003	138	ribonucleotide reductase stimulatory protein	69.3/30.7	T/C	-
SNP	100650	139		67.9/32.1	T/C	-
SNP	100656	139		68.6/31.4	G/A	-
SNP	100767	139		68.2/31.8	C/T	-
SNP	101101	139		67.2/32.8	A/G	Met -> Val
SNP	101158	139		68.3/31.6	A/G	Asn -> Asp
SNP	101211	139		69.2/30.8	C/T	-
SNP	101223	139		70.9/29.0	A/G	-
SNP	101238	139	ribonucleotide reductase large subunit	69.7/30.3	G/C	-
SNP	101367	139		69.4/30.6	T/C	-
SNP	101406	139		69.9/30.1	T/C	-
SNP	101409	139		68.7/31.3	C/T	-
SNP	101439	139		70.5/29.5	A/G	-
SNP	101628	139		66.1/33.9	A/G	-
SNP	101676	139		65.9/34.1	A/G	-
SNP	101748	139		66.8/33.2	C/T	-
SNP	101775	139		66.9/33.1	C/T	-
SNP	102646	140		68.4/31.6	T/G	-
SNP	102658	140		69.9/30.1	T/C	-
SNP	102691	140	ribonucleotide reductase small subunit	68.8/31.2	C/T	-
SNP	102695	140		69.4/30.6	C/T	-
SNP	102712	140		68.9/31.1	T/A	-
SNP	103518	141	hypothetical protein	69.2/30.8	G/T	Lys -> Asn
SNP	115997	156	tail protein	68.8/31.2	T/C	Val -> Ala
SNP	121244	166	hypothetical protein	64.1/35.9	T/C	-

Table S3. Continued.

SNP	123549	168	putative structural protein	65.7/34.3	A/G	Ile -> Met
SNP	127487	-	-	72.4/27.6	G/A	-
SNP	127489	-	-	72.2/27.8	T/G	-
SNP	127501	-	-	72.4/27.6	C/T	-
SNP	127909	182		76.7/23.3	G/A	Ser -> Asn
SNP	127912	182		79.1/20.9	T/C	Phe -> Ser
SNP	127937	182		78.9/21.1	C/A	Asp -> Glu
SNP	127940	182		78.2/21.8	T/C	-
SNP	127942	182		78.4/21.6	T/C	Val -> Ala
SNP	127950	182		76.8/23.2	G/A	Val -> Ile
SNP	127958	182		76.3/23.7	T/C	-
SNP	127991	182		76.4/23.6	T/A	-
SNP	127992	182		77.2/22.8	G/A	Val -> Ile
SNP	127997	182	hypothetical protein	77.1/22.9	G/A	-
SNP	128025	182		78.0/22.0	T/C	-
SNP	128036	182		78.3/21.7	C/T	-
SNP	128055	182		79.4/20.6	A/G	Met -> Val
SNP	128057	182		77.4/22.6	G/A	Met -> Ile
SNP	128058	182		77.6/22.4	G/A	Gly -> Ser
SNP	128059	182		77.6/22.4	G/A	Gly -> Asp
SNP	128061	182		77.0/23.0	C/A	Gln -> Lys
SNP	128064	182		78.8/21.2	T/C	-
SNP	128075	182		77.8/22.2	T/C	-
SNP	128095	-	-	77.1/22.9	G/A	-
SNP	128098	-	-	76.6/23.4	A/G	-
SNP	128103	183		76.8/23.2	T/C	-
SNP	128115	183		75.8/24.2	T/C	-
SNP	128123	183		75.3/24.7	T/C	-
SNP	128124	183		75.0/25.0	T/C	-
SNP	128126	183	hypothetical protein	76.2/23.8	A/G	-
SNP	128144	183		73.4/26.6	T/C	-
SNP	128156	183		72.7/27.3	C/T	-
SNP	128174	183		72.8/27.2	T/C	-
SNP	128177	183		71.8/28.1	T/A	-
SNP	129292	186	hypothetical protein	69.4/30.6	G/A	Gly -> Ser
DIP	129457^129458	186		70.2/29.8	-/A	frameshift
DIP	131949-134006	195-201	hypothetical proteins and phosphoesterase		deletion[1]	
DIP	131960^131961	195	hypothetical protein	73.3/26.7	-/A	frameshift
SNP	134001	201	hypothetical protein	58.4/41.6	A/T	-
SNP	140140	214		75.8/24.2	T/C	-
SNP	140146	214		74.7/25.3	G/A	-
SNP	140161	214	hypothetical protein	77.0/23.0	C/A	-
SNP	140164	214		77.2/22.8	G/A	-
SNP	140170	214		77.7/22.3	A/T	-
SNP	140173	214		77.3/22.7	T/C	-

[1]polymorphism proved by sequencing of correspondent PCR amplicon

Table S4. Genome differences between phages 812h1 and 812.

812h1				phage 812 ORF	aa identity	fixation of SNPs detected in 812 with impact on encoded protein sequences of 812h1 (position and frequency of occurrence in 812h1 genome)
ORF	strand	position	product			
orf3	+	1093 - 1383	terminal repeat-encoded protein	-	-	
orf4	+	1383 - 1670	terminal repeat-encoded protein	*orf3*	78%	
orf5	+	1670 - 1963	terminal repeat-encoded protein	*orf5*	99%	
orf7	+	2302 - 2541	terminal repeat-encoded protein	*orf7*	99%	
orf20	+	7740 - 8009	terminal repeat-encoded protein	*orf20*	93%	
orf22	-	8639 - 8875	hypothetical protein	*orf22*	99%	
orf23	-	8877 - 9362	hypothetical protein	*	*	
orf24	-	9375 - 9782	hypothetical protein	*	*	
orf25	-	9782 - 9952	hypothetical protein	*orf23*	98%	
orf29	-	10882 - 11313	hypothetical protein	*orf27*	99%	
orf33	-	12777 - 13484	serine/threonine protein phosphatase	*orf31*	99%	
orf36	-	15457 - 16005	hypothetical protein	*orf34*	98%	
orf39	-	16412 - 17149	hypothetical protein	*orf37*	p	G -> A (pos. 17077, f = 100 %)
orf41	-	17328 - 17567	hypothetical protein	*orf39*	99%	
orf42	-	17569 - 17958	transglycosylase	*orf40*	99%	
orf50	-	21180 - 22298	AAA family ATPase	*orf48*	77%	
				orf49	23%	
orf54	-	23621 - 23809	hypothetical protein	*orf53*	98%	
orf56	-	24014 - 26062	hypothetical protein	*orf55*	92%	G -> C (pos. 24643, f = 100 %), C -> T (pos. 24682, f = 100 %), A -> C (pos. 25693, f = 70.3 %)
orf59	-	26600 - 27178	hypothetical protein	*orf58*	p	C -> T (pos. 26605, f = 69.2 %)
orf87	+	44631 - 45431	putative virion protein	*orf86*	99%	
orf90	+	46109 - 47302	hypothetical protein	*orf89*	p	- -> CCTGAA (47054 - 47059, f = 100 %)
orf99	+	54578 - 55456	putative neck protein	*orf98*	99%	
orf120	+	77231 - 80290	tail fiber protein complex	*orf119*	p	A -> C (pos. 79614, f = 100 %)
orf124	+	84609 - 86531	receptor-binding protein	*orf123*	99%	A -> T (pos. 86436, f = 100 %)
orf126	+	86935 - 88311	receptor-binding protein	*orf125*	p	G -> A (pos. 88174, f = 54.8 %)
orf134	+	97234 - 98301	DNA primase	*orf133*	99%	
orf136	+	98706 – 99158	hypothetical protein	*orf135*	p	T -> G (pos. 98832, f = 68 %)
orf139	+	100177 – 102291	ribonucleotide reductase large subunit	*orf138*	p	G -> A (pos. 101101, f = 67.2 %), G -> A (pos. 101158, f = 68.3 %)
orf141	+	103372 – 103701	hypothetical protein	*orf140*	p	T -> G (pos. 103518, f = 69.2 %)
orf142	+	103685 – 104005	oxidoreductase	*orf141*	p	G -> A (pos. 103935, f = 100 %)
orf155	+	115561 – 115677	putative capsid component (truncated)	*orf154*	p	C -> A (pos. 115677, f = 100 %)
orf156	+	115762 – 116082	putative capsid component (truncated)			
orf168	+	123130 - 123588	putative structural protein	*orf167*	99%	
orf174	+	125671 - 125835	membrane-associated protein (truncated)	*orf173*	29%	
orf175	+	125822 - 126001	membrane-associated protein (truncated)		30%	
orf182	+	127719 - 128087	hypothetical protein	*orf180*	90%	
orf186	+	129115 – 129465	hypothetical protein	*orf184*	p	A -> G (pos. 129292, f = 69.4 %)
orf210	+	137545 – 139014	nicotinamide phosphoribosyltransferase	*orf208*	p	
orf211	+	139093 - 139338	hypothetical protein	*orf209*	82%	
orf212	+	139358 - 139750	hypothetical protein	*orf210*	90%	
orf213	+	139752 - 139973	hypothetical protein	*	*	
orf214	+	140039 - 140350	hypothetical protein	*orf213*	86%	
orf215	+	140353 - 140862	hypothetical protein	*	*	
orf216	+	140864 - 141193	hypothetical protein	*	*	
orf217	+	141199 - 141393	hypothetical protein	*orf216*	88%	
orf218	+	141417 - 141731	hypothetical protein	*	*	
orf219	+	141746 - 141913	hypothetical protein	*	*	

Most of the phage 812h1 proteins are identical (not shown in the table) or identical to minor polymorphic variants (p) of phage 812. Sequences of phage 812h1 *orf3* , *orf23* , *orf24* , and *orf211* – *orf219* were not found in the assembled genome of phage 812 but except for *orf3* (indicated by a dash), their sequences were found in phage 812 sequencing data with very poor coverage (indicated with an asterisk). The *orf3-4* of both phages are included in region bordered by the motif AAAGGAGAGAT(T)ATAATGAT. Some of the genes are truncated because of the premature stop codon due to a nonsense mutation (*orf49* of phage 812) or extensive intrinsic deletion (*orf174* of phage 812h1). The SNP located in *orf154* of phage 812 leads to a truncated gene for putative capsid component (variant *orf155*) due to a premature stop codon in minor genomic variants. This SNP was fixed in phage 812h1. EMBOSS Needle was used for global alignment of the sequences along their entire length.

Table S5. Relative quantification of plasmid- and chromosome-borne genes *blaZ* , *tetK* and *mecA* in virions of *Kayvirus* phages K, 812K1/420, PyoPhage and *Phietavirus* phage 80α by qPCR (A) and used primers (B).

A

plasmid-borne gene *blaZ*			
	Bacterial strains		
Bacteriophage	COL	USA300 08/019	RN4220 (pT181, pUSA-Houmr-like)
K	6.87E-06 (0.10E-06; 19.43E-06)	7.38E-07 (0.52E-07; 23.28E-07)	7.55E-07 (0.68E-07; 12.79E-07)
812K1/420	5.72E-07 (0.71E-07; 10.72E-07)	6.13E-06 (5.76E-06; 6.51E-06)	3.98E-07 (2.45E-07; 5.51E-07)
PyoPhage	4.32E-07 (1.48E-07; 7.17E-07)	2.46E-06 (0.40E-06; 12.33E-06)	1.30E-06 (0.12E-06; 3.71E-06)
80α	4.99E-04 (0.10E-04; 12.44E-04)	5.14E-05 (0.38E-05; 12.66E-05)	1.75E-03 (0.74E-03; 5.24E-03)
kayviruses vs. phietavirus 80α (p-value)	**<0.001**	**<0.001**	**<0.001**
phietavirus 80α vs. kayviruses (p-value)	**<0.001**	**<0.001**	**<0.001**
plasmid-borne gene *tetK*			
	Bacterial strains		
Bacteriophage	COL	USA300 08/019	RN4220 (pT181, pUSA-Houmr-like)
K	1.67E-05 (0.73E-05; 2.61E-05)	7.80E-05 (0.89E-05; 34.50E-05)	1.32E-05 (0.59E-05; 3.24E-05)
812K1/420	2.23E-05 (0.71E-05; 6.16E-05)	1.64E-05 (0.33E-05; 5.62E-05)	1.94E-05 (0.94E-05; 2.94E-05)
PyoPhage	1.80E-05 (0.54E-05; 6.14E-05)	1.51E-06 (0.63E-06; 4.65E-06)	1.69E-05 (0.67E-05; 6.06E-05)
80α	6.70E-03 (0.87E-03; 17.27E-03)	2.38E-03 (0.23E-03; 5.98E-03)	4.72E-03 (0.83E-03; 12.27E-03)
kayviruses vs. phietavirus 80α (p-value)	**<0.001**	**<0.001**	**<0.001**
phietavirus 80α vs. kayviruses (p-value)	**<0.001**	**<0.001**	**<0.001**
chromosome-borne gene *mecA*			
	Bacterial strains		
Bacteriophage	COL	USA300 08/019	RN4220 (pT181, pUSA-Houmr-like)
K	1.43E-06 (0.23E-06; 5.10E-06)	1.65E-07 (0.56E-07; 4.87E-07)	NR
812K1/420	3.62E-07 (1.61E-07; 5.63E-07)	8.78E-07 (14.30E-07; 31.85E-07)	NR
PyoPhage	6.15E-08 (1.33E-08; 11.77E-08)	4.79E-08 (0.19E-08; 11.77E-08)	NR
80α	7.55E-04 (0.43E-04; 23.53E-04)	4.63E-05 (0.59E-05; 14.84E-05)	NR
kayviruses vs. phietavirus 80α (p-value)	**<0.001**	**<0.001**	NR
phietavirus 80α vs. kayviruses (p-value)	**<0.001**	**<0.001**	NR

B

Primer name	Sequence	Target	Reference
blaZ-F	ACGAAATCGGTGGAATCAAA	*blaZ; S. aureus* USA300_TCH1516 plasmid pUSA300-HOUMR (CP000731)	Mašlaňová *et al* ., 2013[69]
blaZ-R	AGCAGCAGGCGTTGAAGTAT		
tetK-F	ATCTGCTGCATTCCCTTCAC	*tetK; S.aureus* COL plasmid pT181 (CP000045)	
tetK-R	TCCCCCTATTGAAGGACCTAA		
mecA-1575	AGGTTACGGACAAGGTGAAATACTG	*mecA; Staphylococcus aureus* COL (CP000046)	
mecA-1657	TGTCTTTTAATAAGTGAGGTGCGTTAA		
SGB1	ACTTATCCAGGTGGYGTTATT G	tail appendices, phage 80α (NC_009526) - reference gene	Pantůček *et al.* , 2004[44]
SGB2	TGTATTTAATTTCGCCGTTAGTG		
SGD	TGGGCTTCATTCTACGGTGA	*Kayvirus* phage structural protein - reference gene	
SGD	GTAATTTAATGAATCCACGAGAT		

NR - *S. aureus* RN4220 is non-*mecA* strain without SCC*mec* element. p-values were obtained by one-way analysis of variance (ANOVA) on mean values of relative packaging frequency transformed as $\log_{10}$. The relative packaging frequency was calculated as ratio of copy number of target genes and copy number of reference phage borne genes. Mean values of 95% confidence interval are given in brackets. Analyses were performed in statistical software SPSS Statistics v.20 (IBM).

Table S6. Primers and polymerase chain reaction conditions for differentiation of the phage 812-derived mutants: (A) primers, (B) thermal cycling conditions, and (C) expected amplicon size.

A

Name	Sequence	Tm [°C]	CG [%]	nt
812dif1_F	ACAGCATACGTTCTAAAGGAACAA	62.9	37.5	24
812dif1_R	TTCAGTATAGCTCCATCCCGGT	65.9	50.0	22
812dif2_F	TTGCAGATTTAGGATATGCTTCAC	63.3	37.5	24
812dif2a_R	ATGTCTCGTTGTAGTTATTAGCACT	59.4	36.0	25
812dif2b_R	CTGACCAAAATGTACTCATATACCC	61.6	40.0	25
endlys_F	CCATAGAACGGTGAGGACAGG	66.0	57.1	21
endlys_R	TGGAGTGGGTGAGAATCCCTT	67.2	52.4	21

B

Step	Conditions	Cycles
Denaturation	94°C for 30 seconds	1
Denaturation	94°C for 15 seconds	
Annealing	52°C for 20 seconds	25
Extension	68°C for 55 seconds	
Final extension	68°C for 5 minutes	1

C

Combination of primers	Amplicon length [bp]						Targeted gene (phage 812)
	812	812a	812F1	812K1	812K1/420	812h1	
812dif1_F + 812dif1_R	887	887	887	887	887	660	*orf172-orf174*
812dif2_F + 812dif2b_R	-	510	510	510	510	-	*tmpA - orf105*
endlys_F + endys_R	354	354	-	354	-	354	*lysK (orf73)*
812dif2_F + 812dif2a_R	138	-	-	-	-	138	*tmpA (orf103)*
